# SignBase, a collection of geometric signs on mobile objects in the Paleolithic

**DOI:** 10.1038/s41597-020-00704-x

**Published:** 2020-10-23

**Authors:** Ewa Dutkiewicz, Gabriele Russo, Saetbyul Lee, Christian Bentz

**Affiliations:** 1 Department of Early Prehistory and Quaternary Ecology, University of Tübingen, Tübingen, Germany; 2 Department of General Linguistics, University of Tübingen, Tübingen, Germany; 3 DFG Center for Advanced Studies “Words, Bones, Genes, Tools”, University of Tübingen, Tübingen, Germany; 4 Museum für Vor- und Frühgeschichte, Staatliche Museen zu Berlin – Stiftung Preußischer Kulturbesitz, Berlin, Germany

**Keywords:** Archaeology, Evolution of language

## Abstract

In the Paleolithic, geometric signs are abundant. They appear in rock art as well as on mobile objects like artworks, tools, or personal ornaments. These signs are often interpreted as a reflection of symbolic thought and associated with the origin of cognitively modern behavior. *SignBase* is a project collecting the wealth of geometric signs on mobile objects in the European Upper Paleolithic, African Middle Stone Age (MSA), as well as selected sites from the Near East and South East Asia. Currently, more than 500 objects of the Aurignacian techno-complex (ca. 43,000 to 30,000 years BP) are registered in SignBase. They are linked to information about geographic and archaeological provenience, the type of object and material, size and preservation, and respective literature references. We identify around 30 different sign types found on these objects across Europe in the Aurignacian and illustrate how SignBase can be used to analyze geographical clusters. Ultimately, we aim to enable quantitative analyses of abstract graphical expression before the emergence of writing.

## Abstract (summary)

**Table:** 

Measurement(s)	Geometric Shape • geometric signs and motifs • symbol
Technology Type(s)	visual observation method • digital curation
Factor Type(s)	type of mobile object • Paleolithic period

Machine-accessible metadata file describing the reported data: 10.6084/m9.figshare.13033994

## Background & Summary

**Fig. 1:**
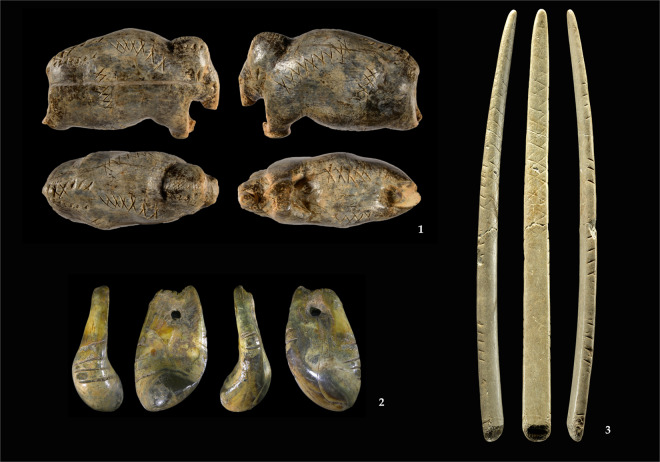
Examples of mobile objects with geometric signs from the Aurignacian. 1. Vogelherd, mammoth figurine, ivory, length 5 cm (Lipták © University of Tübingen); 2. Hohlenstein-Stadel, deer-tooth, personal ornament, size 2.9 cm (Dutkiewicz © Landesamt für Denkmalpflege im Regierungspräsidium Stuttgart); 3. Vogelherd, *lissoir*, bone, size 21 cm (Lipták © University of Tübingen).

In the Paleolithic, geometric signs are found in parietal art as well as on mobile objects. Most of these signs appear in the period between 100,000 and 10,000 BP, but some examples are known from earlier periods^1,2^. The Paleolithic is further subdivided into so-called techno-complexes. For instance, the Aurignacian is an Early Upper Paleolithic techno-complex dating to around 43,000 to 30,000 BP^3–9^. It roughly corresponds to the time when anatomically modern humans migrated into the Near East and Europe and encountered and lived alongside Neanderthals for several millenia. One of the characteristics of the Aurignacian is the abundant use of osseous material for the production of tools, weapons, ornaments, and artworks (see Fig. 1)^10–22^. Many of these objects are decorated with geometric signs.

Geometric signs are sometimes referred to as *abstract motifs*, *patterns*, *marks*, or *jottings* in the literature. We use the term “sign” here in line with Peirce’s semiotics^23,24^, i.e. in the broad sense of a representation of some kind, which is then further subdivided into *index*, *icon*, and *symbol*^10,25,26^. In this context, the term “abstract” is often used to further underline that the signs are not obviously iconic, that is, they cannot be recognized by modern viewers as figurative depictions. Simple geometric forms such as dots, lines, and crosses, as well as more complex patterns such as grids or overlapping crosses, are interpreted in this sense. However, in some cases, even seemingly abstract signs might bear some iconicity. For instance, when applied to animal figurines, simple dots might reflect patterns on fur which were discernible for the Paleolithic viewer. With this caveat in mind, we here choose to use the attribute “geometric” rather than “abstract”. We thus merely refer to the visual property of signs, without further delving into the issue of whether particular signs are to be seen as *indices*, *icons*, or *symbols*.

There are several studies investigating geometric signs in parietal art^27–30^. However, studies scrutinizing signs on mobile objects, such as figurines, tools, or personal ornaments, are rare and mostly limited to either single objects, or to particular assemblages^10,31–36^. SignBase aims to provide extensive data on mobile objects and the geometric signs found on these. A large body of data from the Swabian Aurignacian is available from previous collection efforts^10,31,32^, involving first-hand analyses by the first author. We complement this data with other Aurignacian assemblages available via the literature^3,22,37–55^. We thereby enable quantitative analyses of this rich material.

Early graphical expressions, such as found on objects from the MSA site Blombos in South Africa, have sometimes been interpreted as symbolism and associated with the cognitive modernity of humans, in some cases even with the presence of “fully syntactic language”^56,57^. However, a recent study performing experiments with modern-day participants comes to the conclusion that these engravings – while reflecting “socially transmitted cultural traditions” – bear no clear indication of symbolism^36^. Systematic studies of geometric signs beyond the earliest Paleolithic finds in South Africa will further help to establish their semiotic status. This will also give us a better understanding of their relation to later symbolic behavior such as early writing systems.

Apart from insights into the evolution of human cognition, the geographic distribution of geometric signs – and the associated archaeological cultures – is a second major line of research. Geographic analyses promise to shed light on cultural developments and population turn-overs across the Late Pleistocene, as inferred by the archaeological and human fossil record^58–61^. As an example of practical applications in this direction, we give a preliminary clustering analysis of the sign types found across Europe in the Aurignacian.

## Methods

### Objects

Decorated mobile objects are mostly (though not exclusively) made from osseous material, like ivory, bone, or antler, and usually come from stratified archaeological contexts. In contrast to the chronological difficulties of dating parietal art^62–64^, mobile objects are usually well-dated, at least with reference to the given techno-complex. The data of SignBase is structured according to these archaeological objects. Every artifact that carries geometric signs is assigned an object identifier consisting of a three-letter abbreviation of the excavation site (e.g. Vogelherd Cave: vhc) and a running, four-digit number. The identifier is linked to detailed information about the object. This is firstly the techno-complex, geographic information, stratigraphical unit (layer), and the dating method(s). The year of excavation is also indicated, since very old excavations may lack the relevant information, or it might be inadequate. Secondly, we give information about the object itself: the material, the type of object (object type), the dimensions (length, width, depth) as well as the state of preservation (complete, almost complete, and fragmented). This is followed by a short description of the object, as well as the relevant literature, such as excavation reports. The data file containing all objects and respective information is described in more detail in the section on Data Records. A website displaying this information per object is available online at www.signbase.org.

**Fig. 2:**
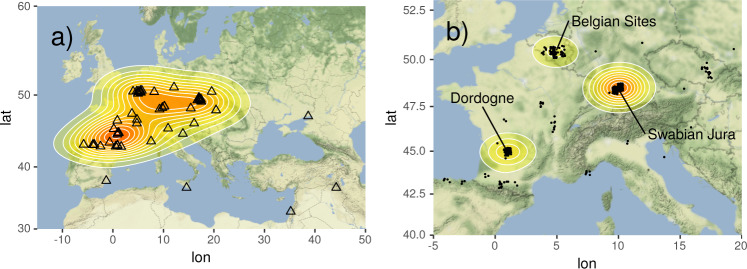
Maps of the Aurignacian sites across Europe and the Near East. (**a**) Each triangle indicates an archaeological site where artifacts carrying geometric signs were found. A density plot is overlaid with high (red) and low (yellow) densities of sites. (**b**) Zoom into the areas yielding most objects with geometric signs. Individual artifacts are plotted as black dots (with some jitter added to avoid overplotting if many artifacts come from the same site). A density plot is overlaid with high (red) and low (yellow) densities of artifacts (for script see Supplementary File 1).

So far, 531 mobile objects carrying geometric signs from 65 Aurignacian sites in Europe and the Near East are registered in SignBase. The locations of excavation sites and individual artifacts can be seen in Fig. 2. The majority of sites that yield artifacts carrying geometric signs of the Aurignacian derive from four main areas: South-West France (in particular the Dordogne), the Swabian Jura in southern Germany, as well as a series of sites in modern-day Belgium, and the Czech Republic. There are further isolated sites in Southern Spain (El Salitre), Sicily (Riparo di Fontana Nuova), Israel (Hayonim cave), Iraq (Shanidar cave), and by the Black Sea (Muralovka) (Fig. 2a). While the density of sites is highest in the Dordogne, the density of artifacts is highest in the Swabian Jura (in particular the Vogelherd cave with overall around 170 artifacts) (Fig. 2b). Of course, both the distribution of sites and the distribution of objects are influenced by historical factors such as excavation and publication efforts of particular universities and researchers. Note, however, that many Aurignacian sites across Europe have not yielded artifacts with geometric signs^22,37^. Hence, while the picture can still change as new sites are discovered and new artifacts published, we expect to have uncovered the main tendencies of the Aurignacian.

The majority (n = 450) of decorated mobile objects from the Aurignacian are made from osseous material (see Table 1). Rock material, like limestone, flint, or other rock types were used in 72 cases. The objects carrying geometric signs are in 430 cases so-called symbolic artifacts (e.g. figurines), tools are present in 72 cases (see Table 2).

**Table 1:** Overview of the raw materials used for objects with geometric signs from the Aurignacian (n = 531).

Material		Number	Total
Osseous	Animal tooth	4	
	Antler	32	
	Bone	202	
	Ivory	210	
	Shell	2	450
Rocks	Calcite	1	
	Limestone	51	
	Flint cortex	10	
	Fossil	1	
	Sandstone	1	
	Slate	2	
	Steatite	1	
	Ochre	3	
	Stone indet.	2	72
Undetermined		9	
Total			**531**

**Table 2:** Overview of the types of Aurignacian objects bearing geometric signs (n = 531).

Object Type		Number	Total
Symbolic	Statuette	40	
	Possible statuette	25	
	Pendant	23	
	Personal ornament	21	
	Flute	9	
	Possible flute	16	
	Tube/flute	24	
	Tube	16	
	Antler object	3	
	Plaquette	4	
	Bloc	47	228
Tools	Awl	21	
	Bâton percé/perforated baton	7	
	Blade	10	
	Compresseur	2	
	Crayon	1	
	Flake	10	
	Lissoir/spatula	43	
	Point	21	
	Rod	32	
	Other	18	165
Undetermined		138	
Total			**531**

### Sign types

**Fig. 3:**
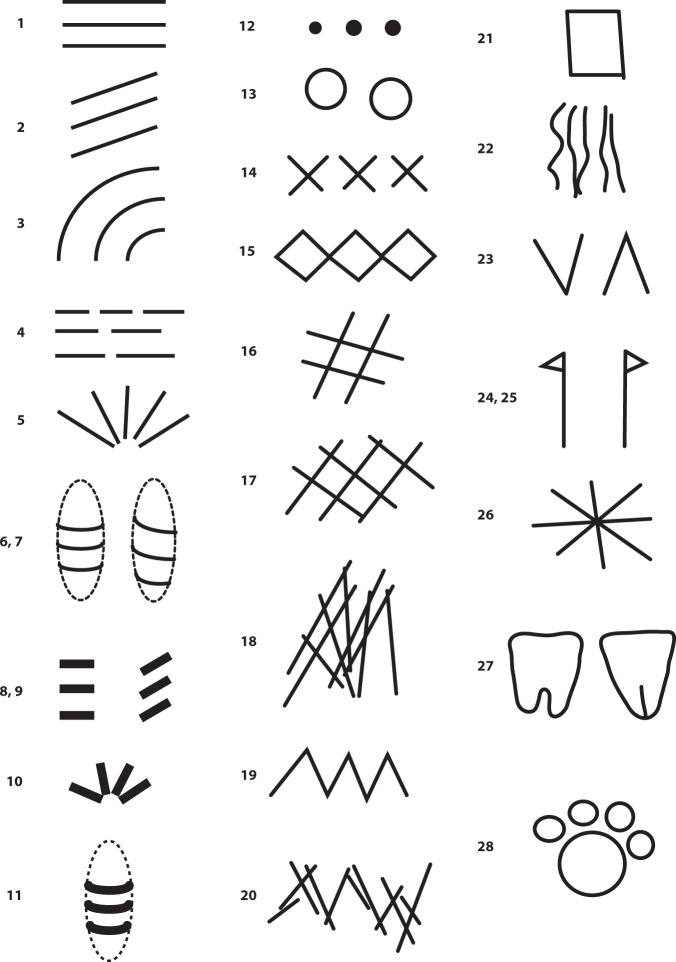
Schematic drawings of sign types as identified for the Aurignacian (in brackets like shown in the data base): 1. Line (line); 2. Oblique line (obline); 3. Concentric lines (concenline); 4. Dashed line (dashline); 5. Radial line (radline); 6. Circumferential line (circumline); 7. Circumferential spiral (circumspiral); 8. Notch (notch); 9. Oblique notch (obnotch); 10. Radial notch (radnotch); 11. Circumferential notch (circumnotch); 12. Dot (dot); 13. Cupule (cupule); 14. Cross (cross); 15. Rhombus (rhombus); 16. Hashtag (hashtag); 17. Grid (grid); 18. Hatching (hatching); 19. Zigzag (zigzag); 20. Zigzag-row (zigzagrow); 21. Rectangle (rectangle); 22. Maccaroni (maccaroni); 23. V-Sign (v); 24. Pin to the left side (pinleft); 25. Pin to the right side (pinright); 26. Star (star); 27. Vulva-Sign (vulva); 28. Paw-Sign (paw). Not shown in the table: anthropomorph, zoomorph, other.

**Fig. 4:**
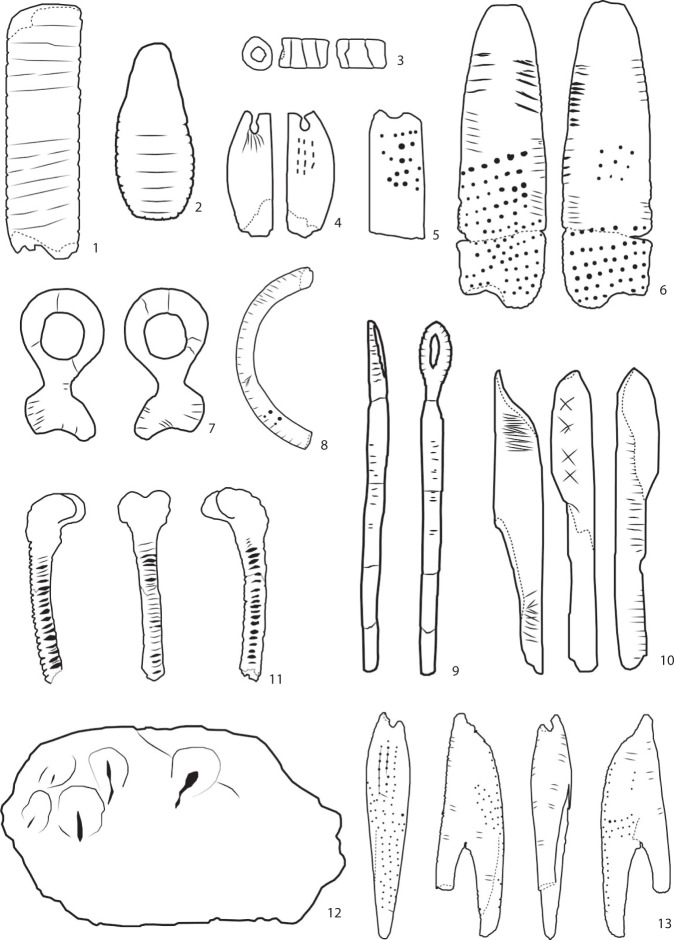
Examples of some Aurignacian mobile objects registered in SignBase and the identified sign types (not in scale, for details see database): 1. aur0001: lines, notches; 2. cas0014: lines, notches; 3. cat0002: circumspiral; 4. gdg0003: dot; 5. bla0018: dots; 6. lar0002: line, obline, notch, dot; 7. gdr0007: line; 8. gpp0004: line, notch, obnotch; 9. msc0005: notch; 10. gdg0008: line, cross, hatching; 11. cel0005: notches; 12. cel0002: vulvae; 13. bla0014: dots, lines.

For each object registered in SignBase we then identify the types of signs represented on it. We define mutually exclusive types, for instance, *straight line*, *oblique line*, *radial line*, *notch*, *dot*, *cross*, and more complex forms like *grid*, *hashtag*, and *zigzag* (see Figs. 3 and 4). Reduced pictograms like *vulvae*, or animal *paws*, are included in this collection as well since they constitute borderline cases between iconic and geometric. For instance, a paw might be a reduced geometric substitute for the entire animal. Overall, we identify 30 different sign types, while uncertain cases are subsumed under the category “other”. Each of the identified sign types is marked as present/absent by one or zero. In Fig. 4, several examples of Aurignacian objects with different sign types are shown, such as *line* (aur0001, cas0014, lar0002, gdr0007, gpp0004, gdg0008, bla0014), *oblique line* (lar0002), *notch* (aur0001, cas0014, lar0002, gpp0005, msc0005, cel0005), *oblique notch* (gpp0004), *circumferential spiral* (cat0002), *dot* (gdg0003, bla0018, lar0002, bla0014), *cross* (gdg0008), *hatching* (gdg0008), and *vulva* (cal0002). Sometimes several sign types appear on a single object. In future versions of the database, numbers of sign occurrences per object, and coding of sign sequences will be included as well.

Note that disagreements between codings by different researchers are unavoidable. The description and typology of geometric signs must be based on visual impressions since we cannot understand the contextual relationships and meanings of such characters from today’s perspective. Any sign typology is hence to some extent subjective and leaves room for discussion. Different sign types can resemble each other, and it is sometimes difficult to determine them undoubtedly. We openly publish our coding decisions and hope for further input and discussion with other researchers. Furthermore, to estimate the degree of subjectivity in our choices, we have submitted the character types defined by us to several peers and calculated agreement scores (see the section on Technical Validation).

### Frequencies of occurrence

**Fig. 5:**
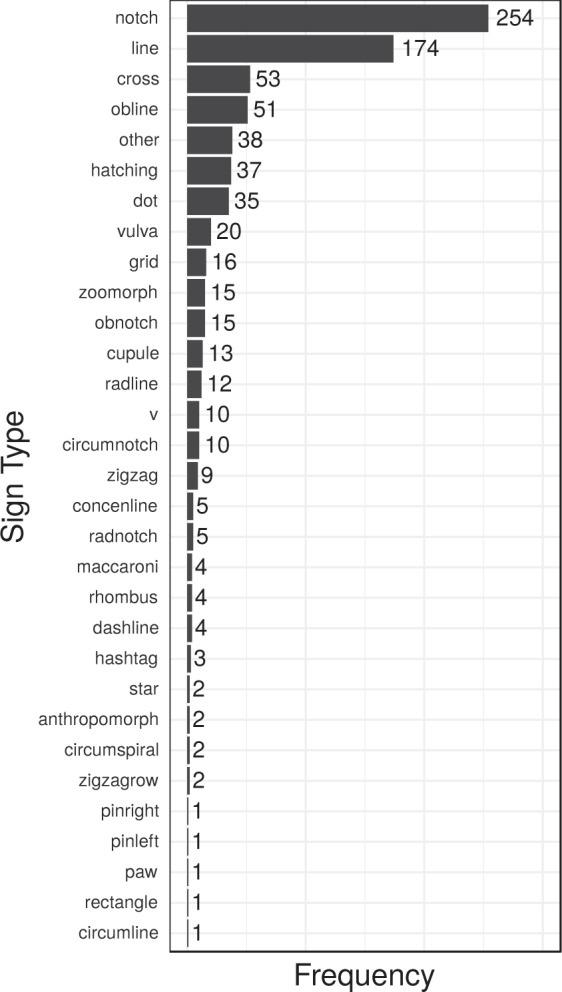
Frequencies of 31 different sign types (including “other”) across the 531 Aurignacian objects (for script see Supplementary File 2).

Given our coding decisions, we can assess how often particular sign types occur. Some are frequently found across different sites and objects, while others are rarer or even restricted to a particular object (see Fig. 5). The most frequent sign type is the simple *notch* (a short incision deeper than a line and in most cases applied on the edge of an object), occurring on almost half of the objects (48%, i.e. 254 of 531), followed by the *line* (33%), and *cross* (10%). *Dots* and *cupules* (7% and 2% respectively) are less frequent but still well-attested, whereas more elaborate signs such as *hashtags*, *stars*, or *zigzags* are exceedingly rare, and often associated with particular objects and sites. For example, clear instances of star-shaped signs (i.e. more than two lines crossing in a center point) are currently only attested on a figurine from the Vogelherd cave (vhc0159), and an engraved ivory blade from the Grotte de La Princesse Pauline in Belgium (gpp0003).

### Geographic clusters

**Fig. 6:**
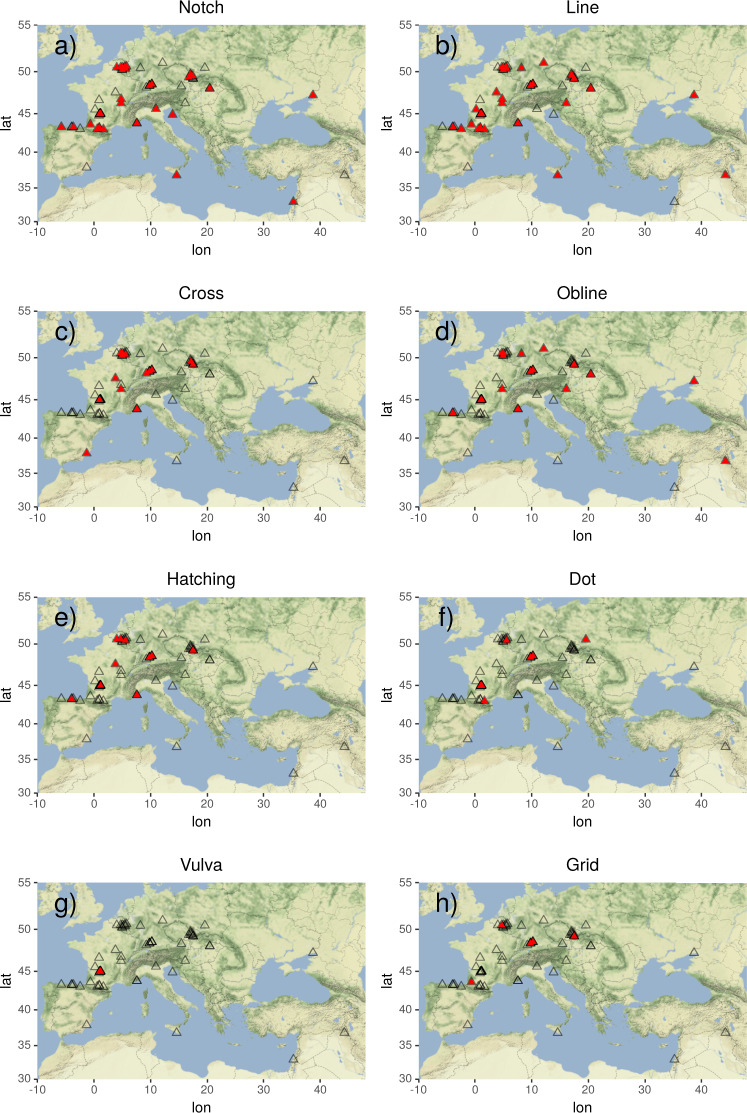
Maps for the presence/absence of particular sign types. Black triangles indicate archaeological sites where artifacts carrying geometric signs were found. Red triangles indicate the presence of a particular sign type (for script see Supplementary File 3).

Apart from differences in frequencies of occurrence, sign types also differ regarding their geographic spread in the Aurignacian. While the most frequent sign types are spread widely across Europe and the Near East, others are geographically more confined (see Fig. 6). The notch, for instance, is ubiquitous. It is found in some of the most westward (e.g. La Viña in Spain) and eastward sites (e.g. Hayonim Cave in Israel), as well as in the most southern (Sicily) and northern sites (Belgium) of central Europe. Others, such as crosses, hatchings, and dots, center around areas of high artifact density such as southwestern France, southern Germany, Belgium, and the Czech Republic. As an extreme example, abstract depictions of vulvae, while being attested a considerable amount of times (i.e. in 20 of 531 objects), are strictly limited to caves in the Dordogne, potentially indicating a local practice of graphic expression^3,37,42,45,50,53,55^.

### Automated analyses

Beyond visual inspection of geographic maps, we here propose a more systematic way of scrutinizing clusters of artifacts and sign types. This also helps to better understand the type of data represented in SignBase. Take the following examples of binary sign type vectors for two objects from the Hohle Fels cave in the Swabian Jura (hfc0015, hfc0006), and Spy in Belgium (spy0023).

hfc0006:                            110100000101000000001000000000                            

*S*^hfc0006^ = {*line*, *oblique line*, *dashed line*, *dot*, *cross*, *v-shape*}

hfc0015:                            111000000100001000000000001000                            

*S*^hfc0015^ = {*line*, *oblique line*, *radial line*, *dot*, *hatching*, *concentric line*}

spy0023:                            100000000001000010001000000000                            

*S*^hfc0015^ = {*line*, *cross*, *zigzag row*, *v-shape*}

These vectors have 30 binary values – the value for “other” is discarded here, which leaves us with 516 objects (i.e. 15 objects have only sign type “other”). The values reflect whether a particular sign type is present (1) or absent (0). An equivalent representation is to give the set of sign types present on an object. These sets are displayed below the binary vectors. The Jaccard distance^65^ between any given two sets *A* and *B* is then calculated as

$${d}_{Jaccard}=1-\frac{\left|A\cap B\right|}{\left|A\cup B\right|},$$

where the numerator is the cardinality of the intersection of the two sets, i.e. the number of shared sign types. Whereas the denominator is the cardinality of the union of two sets, i.e. the overall number of different sign types occurring on both objects together. Thus, the Jaccard distance between hfc0006 and hfc0015 is

$${d}_{Jaccard}^{hfc0006,hfc0015}=1-\frac{3}{9}\approx 0.67.$$

While the Jaccard distance between hfc0006 and spy0023 is

$${d}_{Jaccard}^{hfc0006,spy0023}=1-\frac{3}{7}\approx 0.57,$$

and for hfc0015 and spy0023 we have

$${d}_{Jaccard}^{hfc0015,spy0023}=1-\frac{1}{9}\approx 0.89.$$

Note that while hfc0006 shares three sign types with both hfc0015 and spy0023, the Jaccard distance measure “penalizes” the fact that there are overall more sign types occurring in hfc0006 and hfc0015 together (nine), compared to hfc0006 and spy0023 together (seven). Thus, the Jaccard distance is higher for the former. The rationale behind this is that if there are many different types occurring in two vectors, then it is more likely that the same types occur in both by chance.

Based on pairwise Jaccard distances we create a distance matrix for these three objects as below

$${D}_{Jaccard}=\left(\begin{array}{ccc}0 & 0.67 & 0.57\\ 0.67 & 0 & 0.89\\ 0.57 & 0.89 & 0\end{array}\right).$$

We can then use this distance matrix for cluster analysis.

### Building a UPGMA tree

We here choose the so-called *Unweighted Pair Group Method with Arithmetic mean* (UPGMA) to create a clustering tree. UPGMA is an agglomerative bottom-up clustering method^66^. In the beginning, each “leaf” (object in our case) constitutes its own cluster. Given a distance matrix between clusters, the two clusters with the smallest distance are merged to yield a new cluster. The average distance of this cluster to all other clusters is computed and compared to distances between the other clusters to decide the next merger. The UPGMA algorithm thus successively merges clusters, until only one overall cluster (i.e. the final tree) is formed. Importantly, we here merely use this method to visualize the clustering of objects based on the Jaccard distances of their sign type presences. We do not claim that this clustering reflects actual evolutionary relationships between objects and their sign types. For further details on calculating Jaccard distances and building UPGMA trees see the R code in the files Supplementary File 4 as well as Supplementary File 5.

**Fig. 7:**
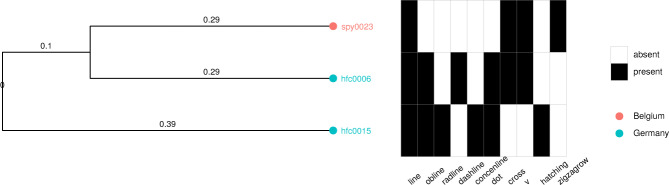
Example of a UPGMA tree for three objects. The object identifiers are given on the tips of branches. Colors indicate the country of provenience. A heatmap is given on the right of the tree with black indicating the presence of a sign type, and white indicating its absence for a given object. Only the sign types which occur in at least one of the three objects are considered here (for script see Supplementary File 4).

Given the Jaccard distance matrix of our three example objects, the UPGMA method yields the tree in Fig. 7. In this simple example, the object from Belgium (spy0023) is first merged with one of the objects from Hohle Fels in Germany (hfc0006), since they have the lowest distance in terms of sign type presence (0.57). The second object from Hohle Fels (hfc0015) is then joined with them in the second step, yielding the overall tree.

**Fig. 8:**
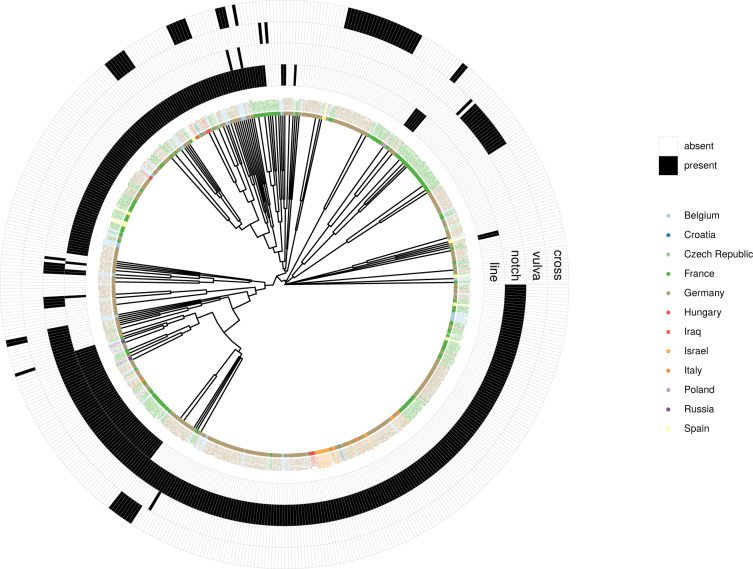
UPGMA tree for 516 Aurignacian objects and sign types. This tree is based on Jaccard distances of sign type presences/absences between pairs of objects. Only some sign types are represented in the presence/absence heatmap around the tree tips (line, notch, vulva, cross). With all sign types included, the plot would be too crowded (for code see Supplementary File 5).

The same method is applied to all 516 objects (excluding objects which are coded as displaying only the sign type “other”) and their Jaccard distances to generate the UPGMA tree in Fig. 8. This gives a general impression of how objects from sites in different countries cluster together based on the presence/absence of particular sign types.

The notch, for instance, is not only widespread (as was pointed out with reference to Fig. 6), but it is also often the only sign type present on objects. This is reflected in the large cluster spanning the lower right quarter of the circular tree in Fig. 8. The objects in this cluster come from a wide range of sites – reflected by the different colors representing modern-day countries. A similar picture emerges for the simple line (mainly upper and lower right quarter of the plot). It is also widely represented across sites, and often the only sign type present on an object. For geometric vulva representations, we find the opposite. They are exclusively found in France (Dordogne), and the objects carrying them mainly cluster together (upper right corner of the plot). There are only three objects carrying vulvae that diverge from this cluster since they carry other sign types (e.g. lines and notches) as well. The cross, on the other hand, is represented in various clusters. There is the main cluster of crosses containing mainly objects from Vogelherd Cave (vhc, upper right corner), but there are also other smaller clusters involving objects from France and Belgium.

However, we do here not further delve into the issues of statistical analyses, hypotheses testing, and interpretation of clusters. These are topics for future studies using SignBase.

## Data Records

The data sets used for analyses in this article are available at figshare^67^: Dutkiewicz, Russo, Lee, & Bentz. SignBase: collection and analysis of geometric signs on mobile objects in the Paleolithic. *figshare* 10.6084/m9.figshare.c.4898643 (2020).

The names of data set files are:

- signBase_exampleObjects.csv (figshare title: “Example Objects”)

- signBase_Version1.0.csv (figshare title: “SignBase Main Data File”)

- TestCoding_000.csv (figshare title: “Test Coding (Coder 0)”)

- TestCoding_001.csv (figshare title: “Test Coding (Coder 1)”)

- TestCoding_002.csv (figshare title: “Test Coding (Coder 2)”)

- TestCoding_003.csv (figshare title: “Test Coding (Coder 3)”)

- TestCoding_004.csv (figshare title: “Test Coding (Coder 4)”)

The main data of the current version of SignBase is given in signBase_Version1.0.csv. As the data set is going to grow, newer versions will be available via www.signbase.org. The column names are given in parentheses and described in the following:

- Microsoft Access ID (access_id): This is an internal ID used by the Microsoft Access database that the online version of SignBase is based on. For the general public, this ID is not important.
- Object identifier (object_id): The object ID is created by a three-letter (lower case) abbreviation of the site name, followed by a four-digit running number, e.g. for objects from *La Ferrassie*: *laf0001*, *laf0002*, etc.
- Techno-complex (techno_complex): Entities in prehistory are based on material cultures. An archaeological culture is a recurring assemblage of artifacts from a specific time and place that may constitute the material culture remains of a past human society. For the Paleolithic, there are mostly artifacts made from lithic or organic material. Characteristic artifact types or assemblages of artifacts define the entity, here referred to as techno-complex. This might be, for instance, the Aurignacian, the Gravettian, or the Magdalenian in Europe, or the Middle Stone Age or the Later Stone Age in Africa.
- Site and location (site_name, location, country, longitude, latitude): All the objects in SignBase are finds from archaeological excavations. These may be caves or open-air sites. The archaeological site (site_name) is indicated, as well as the closest community or town (location) and the country. For an exact location, the latitude and longitude are also given.
- Layer (layer): In archaeological excavations, objects are found in units of sediments, which are called stratigraphical units or layers. These units are usually defined by the archaeologists during excavation and give information about the provenience of the object and its assignment to a particular techno-complex.
- Dating method (dating_method): This describes the method that has been used for dating the find. For absolute dating in Prehistory, mainly radiocarbon dating is used (C14). Other often-used methods are Accelerator Mass Spectrometry (AMS), Thermoluminescence (TL), optically stimulated luminescence (OSL), or Uranium–thorium dating.
- Date (date_max-min): The absolute dating (uncalibrated before present/BP) of the object or layer it derives from as given in the literature.
- Excavation year (excavation_year): The year the object was excavated.
- Material (material): The raw material of the object. For the Paleolithic, mostly osseous material, like ivory, bone, or antler is used. Other organic materials, like shells of mollusks, eggshells, or teeth, but also inorganic materials like rocks, pigments, or ceramics might appear. If the material has not been undoubtedly determined, this feature takes the value *undetermined*.
- Type of object (object_type): Typological determination of the object, usually as indicated in the literature, or revised, if needed. Might be tools, personal ornaments, art figurines, or any other type of archaeological object. If the type of object has not been undoubtedly determined, this feature takes the value *undetermined*.
- Length, width, depth (length_mm, width_mm, depth_mm): Gives the dimensions of the object in millimeters. Usually as indicated in the literature.
- Preservation (preservation): The preservation state of the object. *Complete* – the whole object is preserved; *almost complete* – the whole form of the object is preserved, with some damage; *fragmented* – only partly preserved, the original dimension and shape of the object is not preserved. If the preservation has not been undoubtedly determined, this feature takes the value *undetermined*.
- Short description (short_description): This is a very brief (mostly one sentence) impressionistic description of the type of object and – in some cases – the respective signs represented on it. This mostly follows the description authors use in the original articles publishing the object.
- General literature (general_literature): Literature references about the object, its provenience, excavation report, or dating of the object/stratigraphical unit.

## Technical Validation

Establishing a sign type and its presence/absence on a particular object is a non-trivial task. Decisions are based on subjective judgment. We hence expect some disagreement between our “expert” coding and the potential coding of other researchers in the field. To get a first impression of the degree of subjectivity in our coding decisions, we have submitted 30 randomly chosen objects (of the 531 overall objects) to four colleagues at the University of Tübingen, who are familiar with the archaeological material. Familiarity with the objects and the respective literature is necessary since judging surface patterns is not possible without an understanding of their characteristic texture. This is particularly important given that coding decisions have to be taken based on pictures (photographs and/or drawings, in some cases of low resolution). Disturbances of the material due to natural processes (cracks, wholes, bite marks, etc.), as well as functionally motivated adaptations, can be easily misinterpreted as intentional geometric signs by a layperson.

Having said this, we did not explicitly teach the four test coders how to determine sign type presence. We merely provided them with the same interface used by the SignBase team to make coding decisions. In this interface, a picture of the respective object, alongside the meta-information described above, is provided. Furthermore, there are 31 predefined sign types to choose from, including a category “other”, in case none seem to fit. Test coders were instructed to choose the sign types they identify on any given object. While several different sign types can occur on a single object, each sign (i.e. pattern on the object’s surface) should only be associated with one sign type. For example, if a line is visible, the coder must decide whether it is a regular (i.e. straight) line or an oblique line. The same pattern cannot be coded as both.

**Fig. 9:**
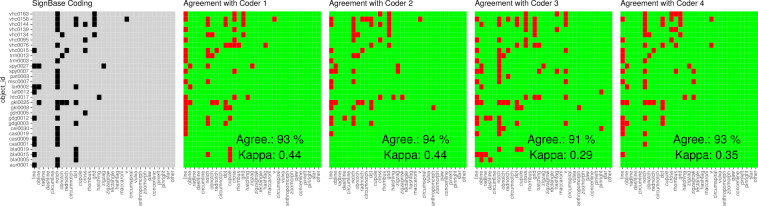
Evaluation of coding. 30 randomly selected objects with object identifiers given on the x-axis. In the left panel, the presence of a given type (y-axis) is given in black, grey tiles indicate the absence of the type according to the original coding by the SignBase team. The plots to the right illustrate the agreement with test coders. Green indicates coding agreement, red indicates disagreement. Percentages of agreeing tiles, as well as Cohen’s Kappas, are given in the lower right corner (for code see Supplementary File 6).

Given the original expert coding by the SignBase team (Test Coding (Coder 0)), and the coding by four test coders (Test Coding (Coder 1), Test Coding (Coder 2), Test Coding (Coder 3), Test Coding (Coder 4)), we firstly calculate the so-called joint-probability agreement, namely, simply the percentage-wise overlap in coding decisions. Secondly, we calculate Cohen’s Kappa^68^. This is a more conservative metric of agreement devised to consider that for any given number of coding decisions, coders might agree just by chance. For example, if coders just choose uniformly at random between presence and absence for all the sign types, the expected agreement of coding decisions between two coders is already 50%. The Kappa metric takes this chance agreement as a baseline. A Kappa value of 0 indicates that there is no coder agreement beyond that predicted by chance, while a Kappa of 1 indicates that there is perfect coder agreement. Additionally, the R package we use to calculate Kappa also provides a p-value. If this p-value is <0.05 for a given Kappa value, then it is significantly bigger than 0. In Fig. 9, the joint-probability agreement (Agree.), as well as Kappa values for pairwise comparisons between the original SignBase coding and the four test coders, is illustrated.

The joint-probability agreement ranges from 91% to 94% between our original coding and the coding by test coders. Cohen’s Kappa ranges from 0.29 to 0.44. While there is hence high coding agreement according to the joint-probability measure, the Kappa values indicate that the agreement between coders is rather moderate. However, given that coders were not explicitly instructed on how to make their decisions, even medium range Kappas are encouraging. The p-values for all Kappas are <0.05 (in fact, they are output as 0), meaning that there is clearly more agreement between coders than expected by chance. The R code with further explanations is given in Supplementary File 6.

## Supplementary information

Supplementary File 1

Supplementary File 2

Supplementary File 3

Supplementary File 4

Supplementary File 5

Supplementary File 6

## Data Availability

The R code to replicate the analyses of this article can be found in a series of R markdown files and at figshare^67^: Dutkiewicz, Russo, Lee, & Bentz. SignBase: collection and analysis of geometric signs on mobile objects in the Paleolithic. *figshare* 10.6084/m9.figshare.c.4898643 (2020). Figure 2: Supplementary File 1 and figshare File 1 Figure 5: Supplementary File 2 and figshare File 2 Figure 6: Supplementary File 3 and figshare File 3 Figure 7: Supplementary File 4 and figshare File 4 Figure 8: Supplementary File 5 and figshare File 5 Figure 9: Supplementary File 6 and figshare File 6

## References

[1] d’Errico F (2003). Archaeological Evidence for the Emergence of Language, Symbolism, and Music - An Alternative Multidisciplinary Perspective. Journal of World Prehistory.

[2] Joordens JCA (2015). Homo erectus at Trinil on Java used shells for tool production and engraving. Nature.

[3] White R (2012). Context and dating of Aurignacian vulvar representations from Abri Castanet, France. Proceedings of the National Academy of Sciences.

[4] Higham T (2012). Τesting models for the beginnings of the Aurignacian and the advent of figurative art and music: The radiocarbon chronology of Geißenklösterle. Journal of Human Evolution.

[5] Conard NJ, Bolus M (2003). Radiocarbon dating the appearance of modern humans and timing of cultural innovations in Europe: new results and new challenges. Journal of Human Evolution.

[6] Conard NJ, Bolus M (2008). Radiocarbon dating the late Middle Paleolithic and the Aurignacian of the Swabian Jura. Journal of Human Evolution.

[7] Jöris, O., Neugebauer-Maresch, C., Weninger, B. & Street, M. The Radiocarbon Chronology of the Aurignacian to Mid-Upper Palaeolithic Transition along the Upper and Middle Danube. In *New Aspects of the Central and Eastern European Upper Palaeolithic – Methods, Chronology, Technology and Subsistence. Symposium by the Prehistoric Commission of the Austrian Academy of Sciences; Vienna, November 9-11, 200* (eds. Neugebauer-Maresch, C. & Owen, L.) vol. 72 (Mitteilungen der Prähistorischen Kommission, 2010).

[8] Jöris O, Street M (2008). At the end of the 14C time scale - the Middle to Upper Palaeolithic record of western Eurasia. Journal of Human Evolution.

[9] Nigst PR (2014). Early modern human settlement of Europe north of the Alps occurred 43,500 years ago in a cold steppe-type environment. Proceedings of the National Academy of Sciences.

[10] Dutkiewicz, E. & Conard, N. J. The symbolic language of the Swabian Aurignacian as reflected in the material culture from Vogelherd Cave (South-West Germany). In *L’art au quotidien. Objets ornées du Paléolithique supérieur. Actes du colloque international Les Eyzies-de-Tayac, 16-20 juin 2014* (eds. Cleyet-Merle, J.-J., Geneste, J.-M. & Man-Estier, E.) vol. numéro spécial 149–164 (2016).

[11] Tartar, E. Origin and Development of Aurignacian Osseous Technology in Western Europe: a Review of Current Knowledge. In *Aurignacian Genius: Art, Technology and Society of the First Modern Humans in Europe. Proceedings of the International Symposium, April 08-10 2013, New York University* (eds. White, R., Bourrillion, R. & Bon, F.) vol. 7 33–55 (2015).

[12] Tartar, E., Teyssandier, N., Bon, F. & Liolios, D. Équipment de chasse, équipment domestique: une distinction efficace? Réflexion sur la notion d’investissement technique dans les industries aurignaciennes. In *Normes techniques et practiques sociales. De la simplicité des outillages pré- et protohistoriques. XXVIe rencontres internationales d’archéologie et histoire d’Antibes* (eds. Astruc, L., Bon, F., Léa, V., Milcent, P.-Y. & Philibert, S.) 107–117 (Éditions APDCA, 2006).

[13] Wolf, S. *Schmuckstücke – Die Elfenbeinbearbeitung im Schwäbischen Aurignacien*. (Kerns Verlag, 2015).

[14] Wolf, S., Münzel, S., Dotzel, K., Barth, M. & Conard, N. J. Osseous Projectiles from the Aurignacian and the Gravettian of the Swabian Jura (Southwest Germany) reflect changing patterns of raw material, technology and typology. In *Osseous Projectile Weaponry: Towards an Understanding of Pleistocene Cultural Variability* (ed. Langley, M. C.) vol. VERT Series 71–87 (Springer, 2016).

[15] Bolus M, Conard NJ (2006). Zur Zeitstellung von Geschossspitzen aus organischen Materialien im späten Mittelpaläolithikum und Aurignacien. Archäologisches Korrespondenzblatt.

[16] Dutkiewicz E, Wolf S, Floss H, Conard NJ (2018). Les objets en ivoire du Jura souabe. L’Anthropologie.

[17] Tejero, J.-M. & Grimaldi, S. Assessing bone and antler exploitation at Riparo Mochi (Balzi Rossi, Italy): implications for the characterization of the Aurignacian in South-western Europe. *Journal of Archaeological Science* **61**, 59–77 (9).

[18] Julien, M., Baffier, D. & Liolios, D. L’outillage en matières dures animales. In *L’Aurignacien de la Grotte du Renne. Les fouilles d’André Leroi-Gourhan à Arcy-sur-Cure (Yonne)* (ed. Schmider, B.) vol. XXXIVe supplément à Gallia Préhistoire 217–250 (2002).

[19] Liolios, D. Variabilité et caractéristiques du travail des matières osseuses au début de l’Aurignacien: approche technologique et économique. (Université de Paris X-Nanterre, 1999).

[20] Liolios, D. Reflexions on the role of bone tools in the definition of the Early Aurignacian. In *Towards a definition of the Aurignacian. Actes du Symposium de Lisbonne, 25-30 juin 2002* (eds. Bar-Yosef, O. & Zilhão, J.) **vol. 45** (Instituto Portugues de Arqueologia, 2006).

[21] Liolios, D. Les instruments osseux. In *Les Aurignaciens* (ed. Otte, M.) 137–151 (Éditions Errance, 2010).

[22] Hahn J (1977). Aurignacien, das ältere Jungpaläolithikum in Mittel- und Osteuropa..

[23] Peirce, C. S. *Collected Papers of Charles Sanders Peirce*. (Harvard University Press, 1931).

[24] Peirce, C. S. *The Essential Peirce: Selected Philosophical Writings/edited by the Peirce Edition Project*. vol. 2 (1893-1913) (Indiana University Press, 1998).

[25] Sauvet, G. Les signes dans l’art mobilier. In *Les objets au Paléolithique supérieur. Tome 2: les voies de la recherche. Colloque de Foix-Le Mas d’Azil (1987)* (ed. Clottes, J.) 83–99 (Dir. Patrimoine, 1990).

[26] Sauvet, G. Les signes pariétaux. In *L’art pariétal paléolithique. Techniques et méthodes d’étude* (ed. GRAPP) 219–234 (CTHS, 1993).

[27] Breuil, A. H. *Quatre cent siècles d’art pariétal. Les cavernes ornées de l’age du renne*. (Fernand Windels, 1952).

[28] Leroi-Gourhan, A. *Préhistoire de l’art occidental*. vol. 1 (Ed. d’Art Lucien Mazenod, 1965).

[29] Clottes, J. & Lewis-Williams, D. *The Shamans of Prehistory. Trance and Magic in the Painted Caves*. (Harry N. Abrams, 1998).

[30] von Petzinger, G. *The First Signs. Unlocking the Mysteries of the World’s oldest Symbols*. (Atria Paperback, 2016).

[31] Hahn, J. *Kraft und Aggression. Die Botschaft der Eiszeitkunst im Aurignacien Süddeutschlands?* (Verlag Archaeologia Venatoria, 1986).

[32] Dutkiewicz E, Wolf S, Conard NJ (2018). Early symbolism in the Ach and the Lone valleys of southwestern Germany. Quaternary International.

[33] Texier P-J (2013). The context, form and significance of the MSA engraved ostrich eggshell collection from Diepkloof Rock Shelter, Western Cape, South Africa. Journal of Archaeological Science.

[34] Henshilwood CS, d’Errico F, Watts I (2009). Engraved ochres from the Middle Stone Age levels at Blombos Cave, South Africa. Journal of Human Evolution.

[35] Conkey MW (1980). The Identification of Prehistoric Hunter-Gatherer Aggregation Sites: The Case of Altamira [and Comments and Reply]. Current Anthropology.

[36] Tylén K (2020). The evolution of early symbolic behavior in Homo sapiens. Proc Natl Acad Sci USA.

[37] Otte, M. *Le Paléolithique Supérieur Ancien en Belgique*. (Musées Royaux d’art et d’histoire, 1979).

[38] Chollot-Varagnac, M. *Les origines du graphisme symbolique. Essai d’analyse des écritures primitives en Préhistoire*. (Éditions de la Fondation Singer-Polignac, 1980).

[39] *Les chemins de l’art Aurignacien en Europe. Das Aurignacien und die Anfänge der Kunst in Europa. Colloque international, internationale Fachtagung, Aurignac, 16-18 septembre 2005*. (2007).

[40] Bourrillon, R. *et al*. A new Aurignacian engraving from Abri Blanchard, France: Implications for understanding Aurignacian graphic expression in Western and Central Europe. *Quaternary International*10.1016/j.quaint.2016.09.063 (2017).

[41] White, R. Production complexity and standardisation in early Aurignacian bead and pendant manufacture: evolutionary implications. In *The human Revolution. Behavioural and Biological Perspectives in the Origins of Modern Humans* (eds. Mellars, P. & Stringer, C.) 332–390 (Princeton University Press, 1989).

[42] Delluc, B. & Delluc, G. *Les manifestations graphiques aurignaciennes sur support rocheux des environs des Exzies (Dordogne)*. vol. 21 (Éditions du CNRS, 1978).

[43] Knecht, H. & White, R. The Abri Cellier (or La Ruth), Commune de Tursac (Dordogne, France). Results of the 1927 Beloit College Excavations. in *French Paleolithic Collections in the Logan Museum of Anthropology* (eds. Breitborde, L. B. & White, R.) vol. 1 (1992).

[44] San Juan-Foucher, C., Vercoutère, C. & Foucher, P. Parures et objets décorés auriganciens de la Grotte de Gargas (Hautes Pyrénées, France). Schmuck und verzierte Objekte aus dem Aurignacien der Höhle Gargas (Hautes Pyrénées, Frankeich). In *Les chemins de l’art Aurignacien en Europe. Das Aurignacien und die Anfänge der Kunst in Europa. Colloque international, internationale Fachtagung, Aurignac, 16-18 septembre 2005* (eds. Floss, H. & Rouquerol, N.) vol. Cahier 4 89–104 (2007).

[45] Mussi, M., Gioia, P. & Negrino, F. Ten small sites: the diversity of the Italian Aurignacian. In *Towards a Definition of the Aurignacian* (eds. Bar-Yosef, O. & Zilhão, J.) vol. Towards a Definition of the Aurignacian. Trabalhos de Arqueologia 45 (American School of Prehistoric Research/Instituto Português de Arqueologia, 2006).

[46] Bartolomei, G. *et al*. La Grotte de Fumane. Un site aurignacien au pied des Alpes. *Preistoria Alpina - MuseoTridentino di Scienze Naturali* **28** (1992), 131–179 (1994).

[47] d’Errico, F., Julien, M., Liolios, D., Vanhaeren, M. & Baffier, D. Many awls in our argument. Bone tool manufacture and use in the Châtelperronian and Aurignacian levels of the Grotte du Renne at Arcy-sur-Cure. *In The Chronology of the Aurigancian and the Transitional Technocomplexes. Dating, Stratigraphies, Cultural Implications. Proceedings of Symposium 6.I of the XIVth Congress of the UISPP (University of Liège, Belgium, September 2-8, 2001)* (eds. Zilhão, J. & d’Errico, F.) vol. 33 247–270 (Instituto Português de Arqueologia, 2003).

[48] *L’Aurignacien de la Grotte du Renne. Les fouilles d’André Leroi-Gourhan à Arcy-sur-Cure (Yonne)*. vol. XXXIVe supplément à Gallia Préhistoire (CNRS Éditions, 2002).

[49] Zilhão J (2007). The Emergence of Ornaments and Art: An Archaeological Perspective on the Origins of “Behavioral Modernity”. Journal of Archaeological Research.

[50] Valoch K (2010). Paläolithische Archäologie in der ehemaligen Tschechoslovakei und ihr Beitrag zur mitteleuropäischen Forschung. Mitteilungen der Gesellschaft für Urgeschichte.

[51] Lejeune, M. Le Trou Magrite et l’art mobilier aurignacien en Belgique: synthèse et problems. Das Trou Magrite und die aurignacienzeitliche Kleinkunst in Belgien: Synthese und Probleme. In *Les chemins de l’art Aurignacien en Europe. Das Aurignacien und die Anfänge der Kunst in Europa. Colloque international, internationale Fachtagung, Aurignac, 16-18 septembre 2005* (eds. Floss, H. & Rouquerol, N.) vol. Cahier 4 131–144 (2007).

[52] Pirson S (2013). The stratigraphy of Spy cave. A review of the available lithostratigraphic and archaeostratigraphic information. Anthropologica et Praehistorica.

[53] Tejero J-M, Belfer-Cohen A, Bar-Yosef O, Gutkin V, Rabinovich R (2018). Symbolic emblems of the Levantine Aurignacians as a regional entity identifier (Hayonim Cave, Lower Galilee, Israel). Proceedings of the National Academy of Sciences.

[54] Vanhaeren M, d’Errico F (2006). Aurignacian ethno-linguistic geography of Europe revealed by personal ornaments. Journal of Archaeological Science.

[55] Barandiarán, I. & García Diez, M. Les débuts du graphisme paléolithique dans le Nord de la péninsule Ibérique. Die Anfänge graphischer Gestaltung im Paläolithikum des Nordens der Iberischen Halbinsel. In *Les chemins de l’art Aurignacien en Europe. Das Aurignacien und die Anfänge der Kunst in Europa. Colloque international, internationale Fachtagung, Aurignac, 16-18 septembre 2005* (eds. Floss, H. & Rouquerol, N.) vol. Cahier 4 (2007).

[56] Henshilwood CS (2002). Emergence of Modern Human Behavior: Middle Stone Age Engravings from South Africa. Science.

[57] Henshilwood, C. S. & Dubreuil, B. Reading the artefacts: gleaning language skills from the Middle Stone Age in southern Africa. In *The Cradle of Language* (eds. Botha, R. & Knight, C.) vol. 12 41–61 (Oxford University Press, 2009).

[58] Posth C (2016). Pleistocene Mitochondrial Genomes Suggest a Single Major Dispersal of Non-Africans and a Late Glacial Population Turnover in Europe. Current Biology.

[59] Fu Q (2016). The genetic history of Ice Age Europe. Nature.

[60] Rigaud S, d’Errico F, Vanhaeren M (2015). Ornaments Reveal Resistance of North European Cultures to the Spread of Farming. PLOS ONE.

[61] Rigaud S, Manen C, García-Martínez de Lagrán I (2018). Symbols in motion: Flexible cultural boundaries and the fast spread of the Neolithic in the western Mediterranean. PLOS ONE.

[62] González-Sainz C, Ruiz-Redondo A, Garate-Maidagan D, Iriarte-Avilés E (2013). Not only Chauvet: Dating Aurignacian rock art in Altxerri B Cave (northern Spain). Journal of Human Evolution.

[63] Hoffmann DL (2018). U-Th dating of carbonate crusts reveals Neandertal origin of Iberian cave art. Science.

[64] Pettitt P, Pike AWG (2007). Dating European Palaeolithic Cave Art: Progress, Prospects, Problems. Journal of Archaeological Method and Theory.

[65] van der Loo MPJ (2014). The stringdist Package for Approximate String Matching. The R Journal.

[66] Huson, D. H., Rupp, R. & Scornavacca, C. *Phylogenetic Networks: Concepts, Algorithms and Applications*. (Cambridge University Press, 2010).

[67] Dutkiewicz E, Russo G, Lee S, Bentz C (2020). Figshare.

[68] Cohen J (1960). A Coefficient of Agreement for Nominal Scales. Educational and Psychological Measurement.

