# Supplementary material for: SignBase, a collection of geometric signs on mobile objects in the Paleolithic
Source: Sci Data. 2020 Oct 23;7:364. doi: 10.1038/s41597-020-00704-x (PMC7585433; doi:10.1038/s41597-020-00704-x)
Supplement: Supplementary file 1 [file 41597_2020_704_MOESM1_ESM.pdf]

SignBase: Maps for Aurignacian sites

Chris Bentz

July 29, 2020

Load libraries

Load the following packages. If these are not yet installed use install.packages("") to install them.

```
library(ggmap)
library(plyr)
library(gridExtra)
```

Load data

Run this code to load the file with data on Aurignacian objects.

```
objects <- read.csv("Data/signBase_Version1.0.csv")
nrow(objects)
```

```
## [1] 531
```

The number of different objects is given above as the number of rows of the file.

Number of objects per site

Choose subset of columns relevant for object count.

```
objects.short <- objects[, c(3:8)]
```

Remove the duplicated rows.

```
objects.short <- unique(objects.short[order(objects.short$site_name), ])
```

Create data frame with sites and number of object counts.

```
sites <- count(objects$site_name)
colnames(sites) <- c("site_name", "number_of_objects")
```

Merge sites and objects.short

```
sites <- merge(sites, objects.short, by = "site_name")
```

Maps with site locations

Load stamenmap of Europe.

```
euromap <- get_stamenmap(bbox = c(left = -15, bottom = 30, right = 50, top = 60),
                         zoom = 5, maptype = c("terrain-background"), crop = TRUE, messaging = FALSE,
                         urlonly = FALSE, force = FALSE,
                         where = tempdir())
```

ggmap with SITE location densities

Plot a map of Europe with excavation site locations and the densities of these.

```
site.density.euromap <- ggmap(euromap) +
  stat_density2d(data = sites,
                 aes(x = longitude, y = latitude, fill = ..level..),
                 alpha = 0.3, size = 0.2,
                 geom = "polygon") +
  geom_density2d(data = sites,
                 aes(x = longitude, y = latitude), size = 0.3, colour = "white") +
  scale_fill_gradient(low = "yellow", high= "red") +
  geom_point(data = sites,
              aes(longitude, latitude), size = 2, shape = 2) +
  theme(legend.position = "none") +
  geom_text(x = -10, y = 57, label = "a)", size = 7)
site.density.euromap
```

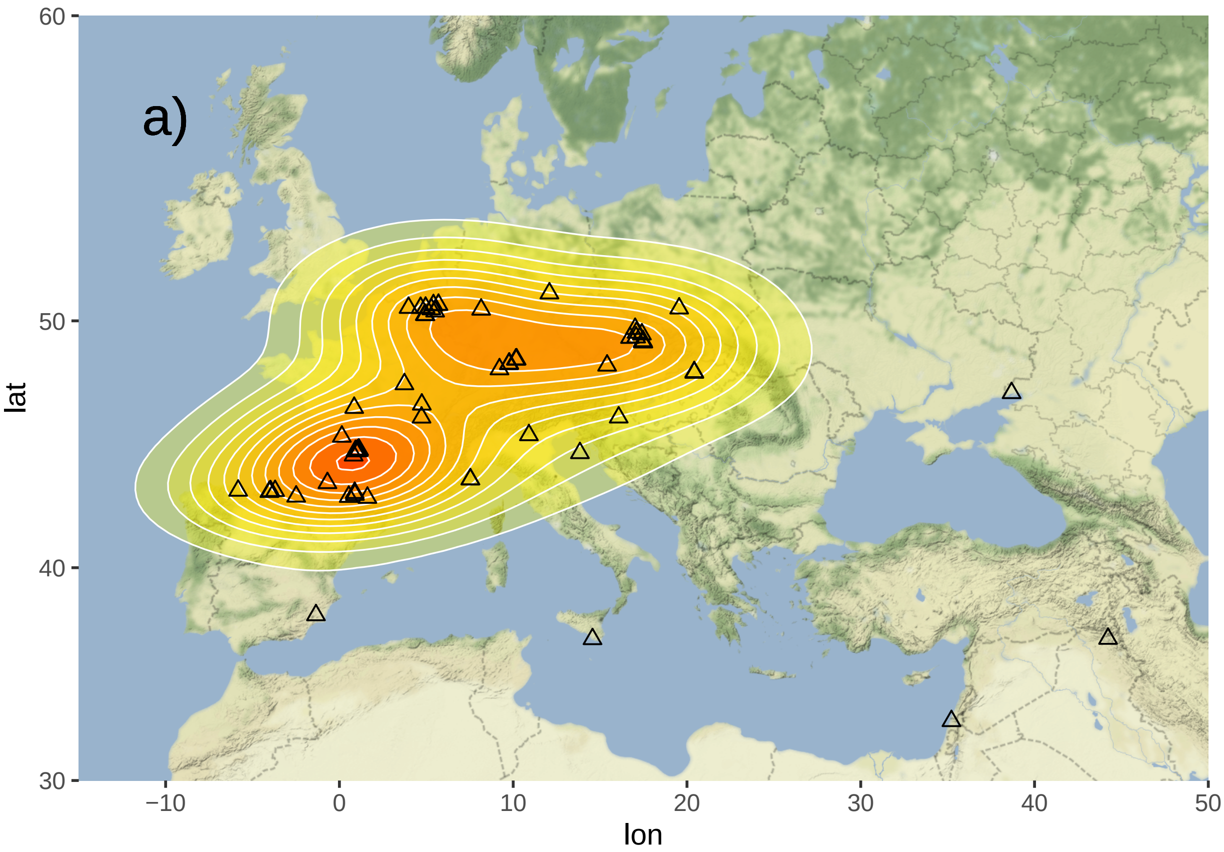

ggmap with OBJECT locations

Load stamenmap of Europe.

```
euromap <- get_stamenmap(bbox = c(left = -5, bottom = 40, right = 20, top = 53),
                         zoom = 5, maptype = c("terrain-background"), crop = TRUE, messaging = FALSE,
                         urlonly = FALSE, force = FALSE,
                         where = tempdir())
```

Plot a map of Europe with each object and its location, and the densities of objects. Note that the object locations need to be jittered, otherwise they would just be overplotted for all objects of the same site.

```
object.density.euromap <- ggmap(euromap) +
  stat_density2d(data = objects,
                 aes(x = longitude, y = latitude, fill = ..level..),
                 alpha = 0.3, size = 0.1,
                 geom = "polygon") +
  geom_density2d(data = objects,
                 aes(x = longitude, y = latitude), size = 0.3, colour = "white") +
  scale_fill_gradient(low = "yellow", high= "red") +
  geom_point(data = objects,
             aes(jitter(longitude, amount = 0.2), jitter(latitude, amount = 0.2)),
             size = 0.1)  +
  theme(legend.position = "none") +
  geom_text(x = -3, y = 51.5, label = "b)", size = 7) +
  annotate("segment", x = 10, xend = 12.2, y = 48.5, yend = 46, colour = "black") +
  annotate("text", x = 16, y = 46, label = "Swabian Jura", colour = "black") +

  annotate("segment", x = 1, xend = -1, y = 45, yend = 47.5, colour = "black") +
  annotate("text", x = -1, y = 48, label = "Dordogne", colour = "black") +

  annotate("segment", x = 5, xend = 6, y = 50.5, yend = 52, colour = "black") +
  annotate("text", x = 9.7, y = 52, label = "Belgian Sites", colour = "black")
object.density.euromap
```

```
## Warning: Removed 19 rows containing non-finite values (stat_density2d).

## Warning: Removed 19 rows containing non-finite values (stat_density2d).

## Warning: Removed 19 rows containing missing values (geom_point).
```

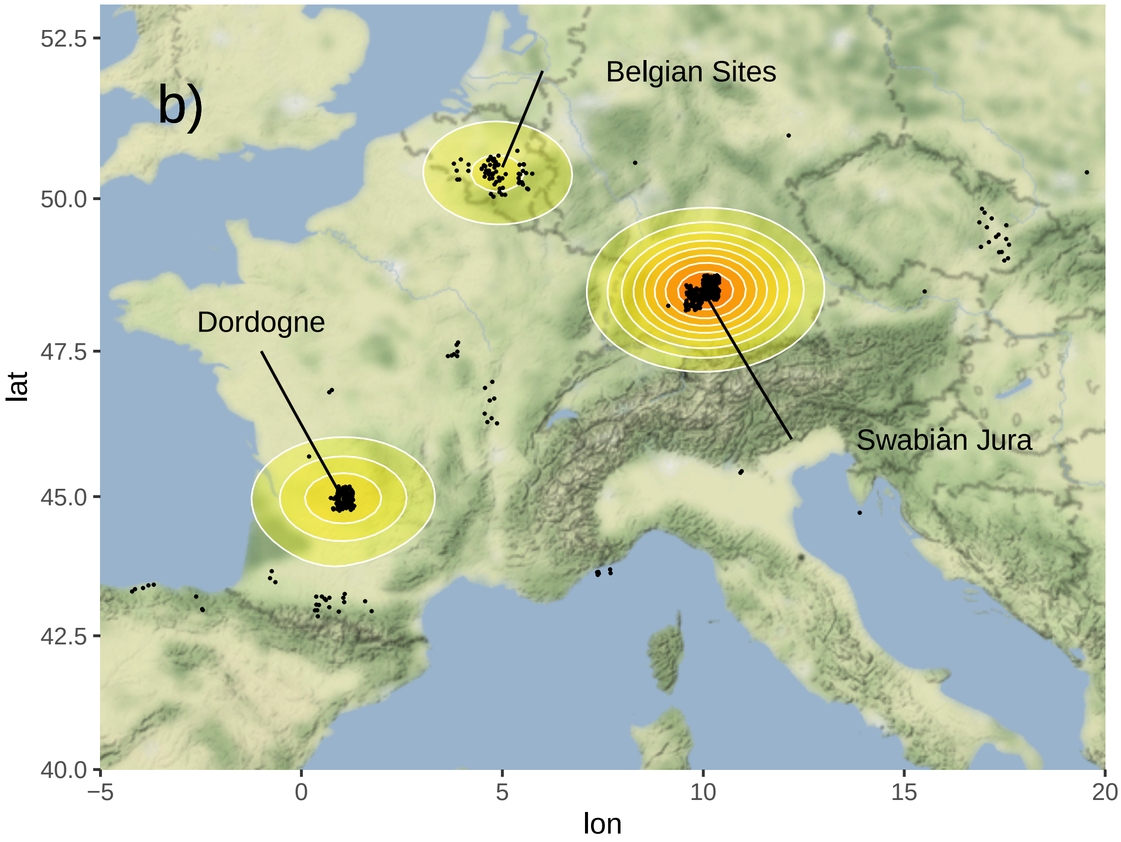

Put both maps together in one panel plot

```
density.maps <- grid.arrange(site.density.euromap, object.density.euromap, ncol = 2)
```

```
## Warning: Removed 19 rows containing non-finite values (stat_density2d).

## Warning: Removed 19 rows containing non-finite values (stat_density2d).

## Warning: Removed 19 rows containing missing values (geom_point).
```

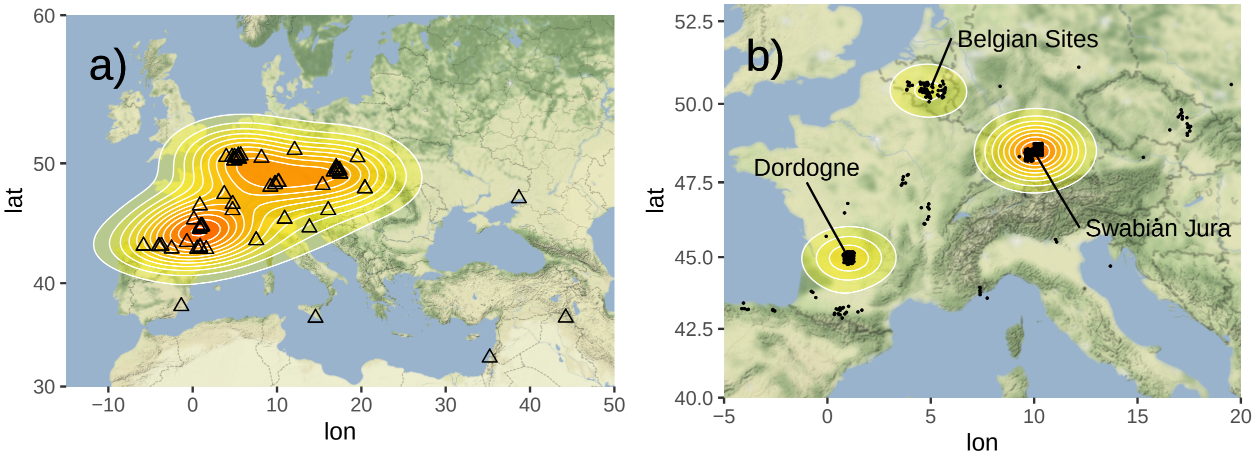

Safe as pdf

Optionally, this plot can be safed to a file as pdf via:

```
ggsave("Figures/Figure_densityMaps.pdf", density.maps, dpi = 300, scale = 1,
       device = cairo_pdf)
```
