# Supplementary material for: SignBase, a collection of geometric signs on mobile objects in the Paleolithic
Source: Sci Data. 2020 Oct 23;7:364. doi: 10.1038/s41597-020-00704-x (PMC7585433; doi:10.1038/s41597-020-00704-x)
Supplement: Supplementary file 2 [file 41597_2020_704_MOESM2_ESM.pdf]

SignBase: Maps for Aurignacian sites with sign type presence/absence

Chris Bentz

July 29, 2020

Load libraries

Load the following packages. If these are not yet installed use install.packages("") to install them.

```
library(ggplot2)
library(ggmap)
library(gridExtra)
library(grid)
```

Load data

Run this code to load the file with data on Aurignacian objects.

```
objects <- read.csv("Data/signBase_Version1.0.csv")
nrow(objects)
```

```
## [1] 531
```

The number of different objects is given above as the number of rows of the file.

Number of objects per site

Choose subset of columns that are relevant for the following analyses.

```
objects.short <- objects[, c(3:8)]
```

Remove the duplicated rows.

```
sites <- unique(objects.short[order(objects.short$site_name), ])
```

Maps with site locations

Load stamenmap of Europe.

```
euromap <- get_stamenmap(bbox = c(left = -10, bottom = 30, right = 48, top = 55),
                         zoom = 5, maptype = c("terrain-background"),
                         crop = TRUE, messaging = FALSE,
                         urlonly = FALSE, force = FALSE,
                         where = tempdir())
```

ggmaps with sign type presence/absence

notch

```
notch.euromap <- ggmap(euromap) +
  geom_point(data = sites,
             aes(longitude, latitude), shape = 2, size = 2, alpha = 0.5) +
  geom_point(data = objects[objects$notch == 1, ],
             aes(longitude, latitude), shape = 17, colour= "red", size = 1.5) +
  ggtitle("Notch") +
  theme(plot.title = element_text(hjust = 0.5)) +
  geom_text(x = -5, y = 53, label = "a)", size = 7)
```

line

```
line.euromap <- ggmap(euromap) +
  geom_point(data = sites,
             aes(longitude, latitude), shape = 2, size = 2, alpha = 0.5) +
  geom_point(data = objects[objects$line == 1, ],
             aes(longitude, latitude), shape = 17, colour= "red", size = 1.5) +
  ggtitle("Line") +
  theme(plot.title = element_text(hjust = 0.5)) +
  geom_text(x = -5, y = 53, label = "b)", size = 7)
```

cross

```
cross.euromap <- ggmap(euromap) +
  geom_point(data = sites,
             aes(longitude, latitude), shape = 2, size = 2, alpha = 0.5) +
  geom_point(data = objects[objects$cross == 1, ],
             aes(longitude, latitude), shape = 17, colour= "red", size = 1.5) +
  ggtitle("Cross") +
  theme(plot.title = element_text(hjust = 0.5)) +
  geom_text(x = -5, y = 53, label = "c)", size = 7)
```

obline

```
obline.euromap <- ggmap(euromap) +
  geom_point(data = sites,
             aes(longitude, latitude), shape = 2, size = 2, alpha = 0.5) +
  geom_point(data = objects[objects$obline == 1, ],
             aes(longitude, latitude), shape = 17, colour= "red", size = 1.5) +
  ggtitle("Obline") +
  theme(plot.title = element_text(hjust = 0.5)) +
  geom_text(x = -5, y = 53, label = "d)", size = 7)
```

hatching

```
hatching.euromap <- ggmap(euromap) +
  geom_point(data = sites,
             aes(longitude, latitude), shape = 2, size = 2, alpha = 0.5) +
  geom_point(data = objects[objects$hatching == 1, ],
             aes(longitude, latitude), shape = 17, colour= "red", size = 1.5) +
  ggtitle("Hatching") +
  theme(plot.title = element_text(hjust = 0.5)) +
  geom_text(x = -5, y = 53, label = "e)", size = 7)
```

dot

```
dot.euromap <- ggmap(euromap) +
  geom_point(data = sites,
             aes(longitude, latitude), shape = 2, size = 2, alpha = 0.5) +
  geom_point(data = objects[objects$dot == 1, ],
             aes(longitude, latitude), shape = 17, colour= "red", size = 1.5) +
  ggtitle("Dot") +
  theme(plot.title = element_text(hjust = 0.5)) +
  geom_text(x = -5, y = 53, label = "f)", size = 7)
```

vulva

```
vulva.euromap <- ggmap(euromap) +
  geom_point(data = sites,
             aes(longitude, latitude), shape = 2, size = 2, alpha = 0.5) +
  geom_point(data = objects[objects$vulva == 1, ],
             aes(longitude, latitude), shape = 17, colour= "red", size = 1.5) +
  ggtitle("Vulva") +
  theme(plot.title = element_text(hjust = 0.5)) +
  geom_text(x = -5, y = 53, label = "g)", size = 7)
```

grid

```
grid.euromap <- ggmap(euromap) +
  geom_point(data = sites,
              aes(longitude, latitude), shape = 2, size = 2, alpha = 0.5) +
  geom_point(data = objects[objects$grid == 1, ],
             aes(longitude, latitude), shape = 17, colour= "red", size = 1.5) +
  ggtitle("Grid") +
  theme(plot.title = element_text(hjust = 0.5)) +
  geom_text(x = -5, y = 53, label = "h)", size = 7)
```

All maps in one panel

```
all.maps <- grid.arrange(notch.euromap, line.euromap, cross.euromap,
                         obline.euromap, hatching.euromap, dot.euromap,
                         vulva.euromap, grid.euromap, ncol = 2)
```

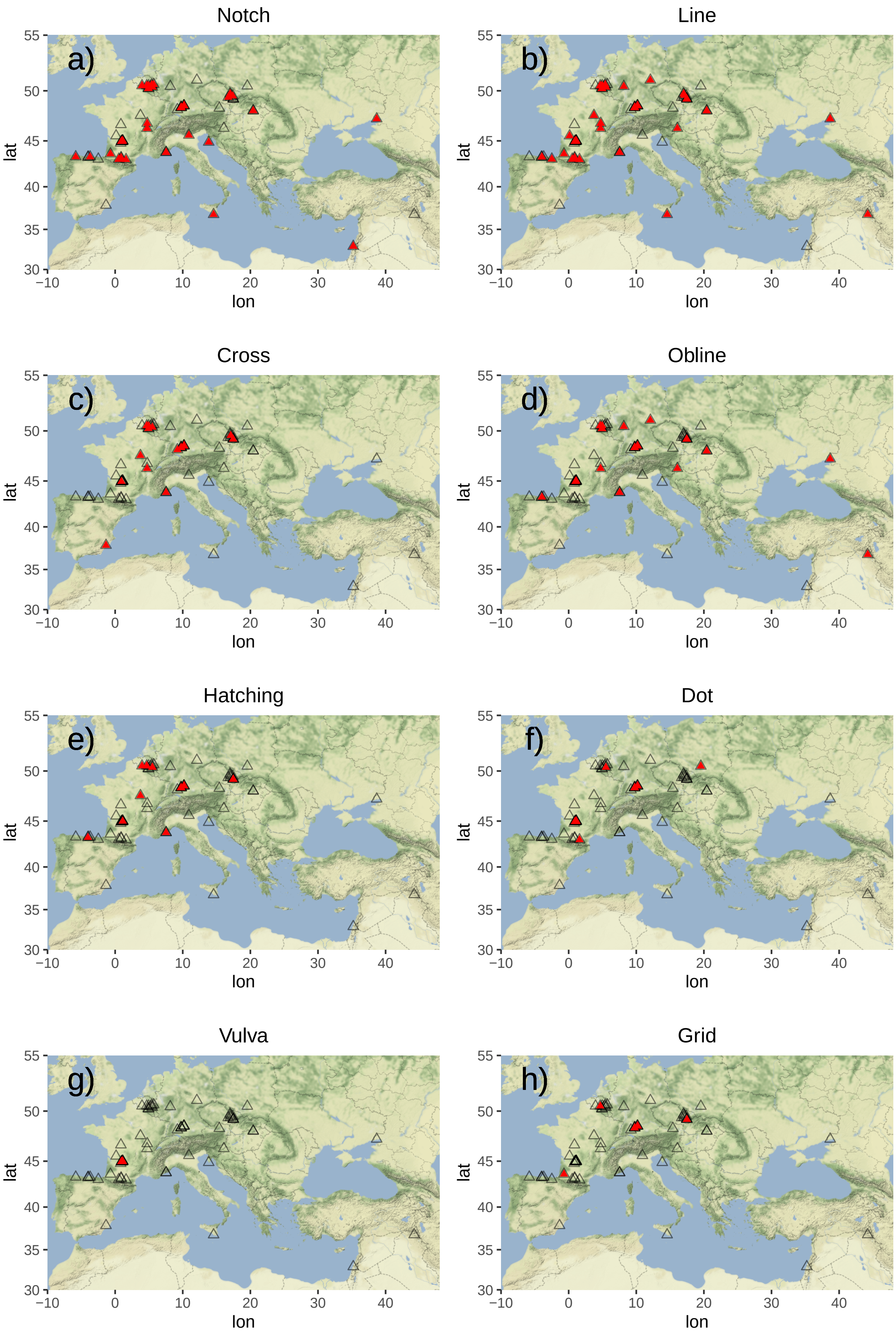
Notch
a)
Line
b)
Cross
c)
Obline
d)
Hatching
e)
Dot
f)
Vulva
g)
Grid
h)
lat
lon
55
50
45
40
35
30
−10
0
10
20
30
40

Safe complete figure to file

```
ggsave("Figures/Figure_signTypeMaps.pdf", all.maps, dpi = 300, scale = 1,
       device = cairo_pdf)
```
