# Supplementary material for: SignBase, a collection of geometric signs on mobile objects in the Paleolithic
Source: Sci Data. 2020 Oct 23;7:364. doi: 10.1038/s41597-020-00704-x (PMC7585433; doi:10.1038/s41597-020-00704-x)
Supplement: Supplementary file 3 [file 41597_2020_704_MOESM3_ESM.pdf]

SignBase: Sign type frequencies for Aurignacian objects

Chris Bentz

July 29, 2020

Load libraries

Load the following packages. If these are not yet installed use install.packages("") to install them.

```
library(ggplot2)
```

Load data

Run this code to load the file with data on Aurignacian objects.

```
objects <- read.csv("Data/signBase_Version1.0.csv")
nrow(objects)
```

```
## [1] 531
```

The number of different objects is given above as the number of rows of the file.

Sign frequency counts

Get the frequencies of occurrence of the different sign types. Therefore, first create empty vectors to be populated.

```
sign.vec <- c()
sign.freq.vec <- c()
```

The following for-loop then counts and appends frequencies of occurrence for each sign type, i.e. from column 21 onwards, which are sign type columns.

```
for (i in 21:ncol(objects)) {
  sign <- colnames(objects)[i]
  sign.vec <- append(sign.vec, sign)
  sign.freq <- sum(objects[, i])
  sign.freq.vec <- append(sign.freq.vec, sign.freq)
}
```

Finally, create a data frame with sign names and respective frequencies.

```
freq.df <- data.frame(sign.vec, sign.freq.vec)
colnames(freq.df) <- c("Sign", "Frequency")
```

Histogram plot

Based on this data frame (freq.df), we can now create a simple histogram with sign type frequencies ordered from highest to lowest.

```
hist <- ggplot(freq.df, aes(x = Sign, y = Frequency))
sign.plot <- hist + geom_bar(stat = "identity") +
  geom_text(aes(label = Frequency), hjust = -0.2, vjust = 0.5,
            position = position_dodge(.9), size = 4, colour = "black") +
  labs(x = "Sign Type", y = "Frequency", size = 3) +
  scale_x_discrete(limits = freq.df[order(freq.df$Frequency),]$Sign) +
  scale_y_continuous(limits = c(0, 300)) +
  coord_flip() +
  theme_bw() +
  theme(axis.title.x = element_text(size = 15),
        axis.title.y = element_text(size = 15),
        axis.text.x = element_blank(), axis.ticks = element_blank())
sign.plot
```

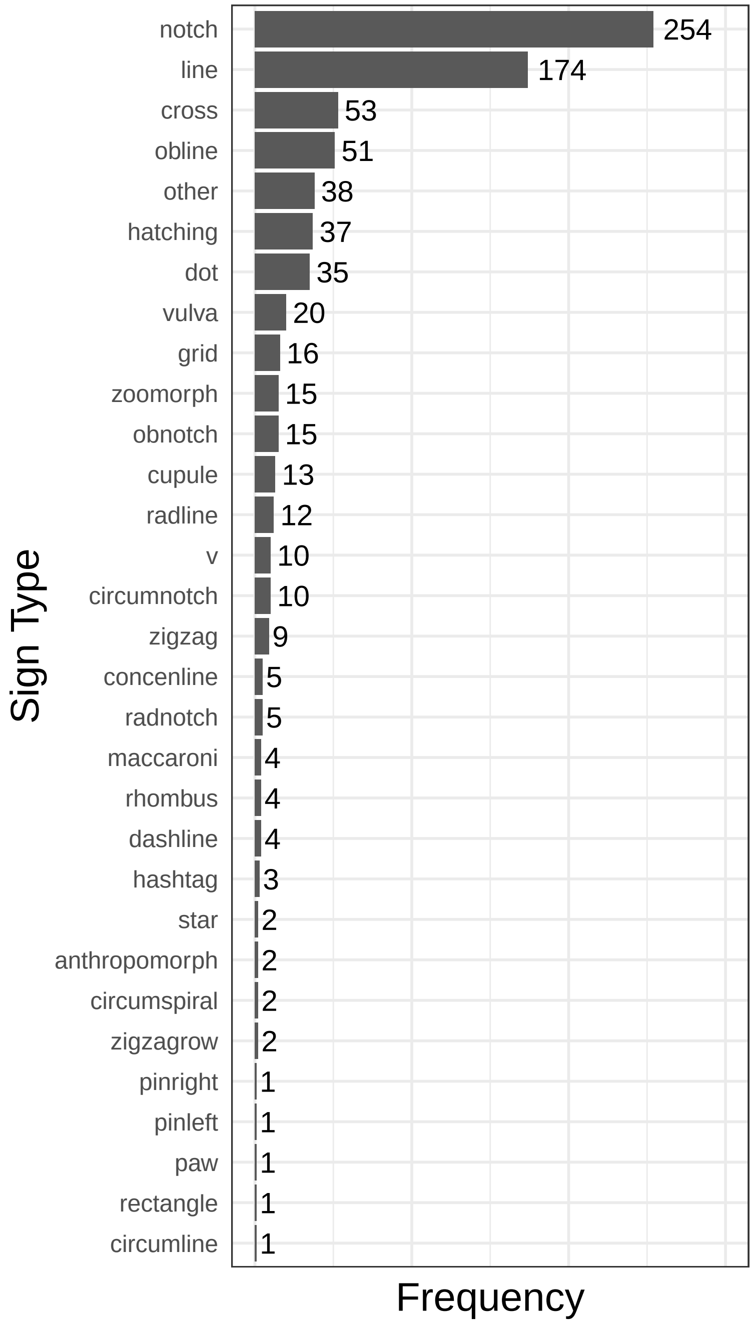

Safe to file

Optionally, this plot can be safed to a file as pdf via:

```
ggsave("Figures/Figure_signTypeFrequency.pdf", sign.plot, dpi = 300, scale = 1, device = cairo_pdf)
```
