# Supplementary material for: SignBase, a collection of geometric signs on mobile objects in the Paleolithic
Source: Sci Data. 2020 Oct 23;7:364. doi: 10.1038/s41597-020-00704-x (PMC7585433; doi:10.1038/s41597-020-00704-x)
Supplement: Supplementary file 4 [file 41597_2020_704_MOESM4_ESM.pdf]

SignBase: UPGMA trees for sign types across Europe

Chris Bentz

July 29, 2020

Load libraries

Load the following packages. If these are not yet installed use install.packages("") to install them.

```
library(stringdist)
library(phangorn)
library(ggtree)
library(RColorBrewer)
library(dplyr)
```

ggtree installation

ggtree is not a standard package at CRAN. In the version of R used here (3.4.4) it has to be installed using the following code.

```
# source("https://bioconductor.org/biocLite.R")
# BiocInstaller::biocLite(c("ggtree"))
```

Load data

Run this code to load the file with data on Aurignacian objects.

```
objects <- read.csv("Data/signBase_Version1.0.csv")
nrow(objects)
```

```
## [1] 531
```

Remove sign type column "other".

```
objects <- subset(objects, select = -c(other))
```

Remove rows with objects that only had "other" as sign type. This leaves 516 objects in the data frame, meaning that 15 objects only had sign type "other".

```
objects$sum <- rowSums(objects[, 21:ncol(objects)]) # add column with sum of 0s and 1s
objects <- objects[objects$sum != "0", ] # remove rows where the sum is 0
objects <- subset(objects, select = -c(sum)) # remove column with sums again
rownames(objects) <- 1:nrow(objects) # change rownames to reflect the new number of rows
nrow(objects)
```

```
## [1] 516
```

Jaccard distance matrix between pairs of objects based on sign type presences

Initialize empty vector.

```
jaccVec <- c()
```

Create an alphabet to represent absence/presence of a given sign type.

```
alphabet <- c("a","b","c","d","e","f","g","h","i","j",
              "k","l","m","n","o","p","q","r","s","t",
              "u","v","w","x","y","z", "A", "B", "C", "D",
              "E", "F", "G", "H", "I", "J", "K", "L", "M", "N")
```

Draw characters from the alphabet to represent given number of sign types.

```
sign.alphabet <- sample(alphabet, ncol(objects[, 21:ncol(objects)]))
```

Use for-loop to replace 1s (sign type present) by respective character. This is necessary to then later apply the function stringdistmatrix() to the strings. There must be a simpler option to compute Jaccard distances directly on binary vectors, but I was not able to find a ready-made and robust function for this.

```
for (i in 1:nrow(objects)) {
  jaccString <- ""
  for (j in 21:ncol(objects)) {
    if (objects[i,j] == 1) {
      jaccString <- paste(jaccString, sign.alphabet[j-20], sep = "")
    } else {
      jaccString <- jaccString
    }
  }
  jaccVec <- append(jaccVec, jaccString)
}
```

Apply Jaccard distance function to create matrix of pairwise distances between objects.

```
signDistMatrix <- stringdistmatrix(jaccVec, jaccVec, method = "jaccard")
```

UPGMA tree based on Jaccard distance matrix

Create UPGMA tree based on the distance matrix based on sign type presences

```
signTree <- upgma(signDistMatrix)
```

Create first column in data frame "signs" with running numbers from 1 to the number of objects. This is needed as ggtree takes the numbers in the first column as identifier for tip values (for whatever reason).

```
tip.id <- 1:nrow(objects)
objects <- cbind(tip.id, objects)
```

Plot ggtree with just tree branches.

```
ggtree.plot <- ggtree(signTree, layout = "circular")
print(ggtree.plot)
```

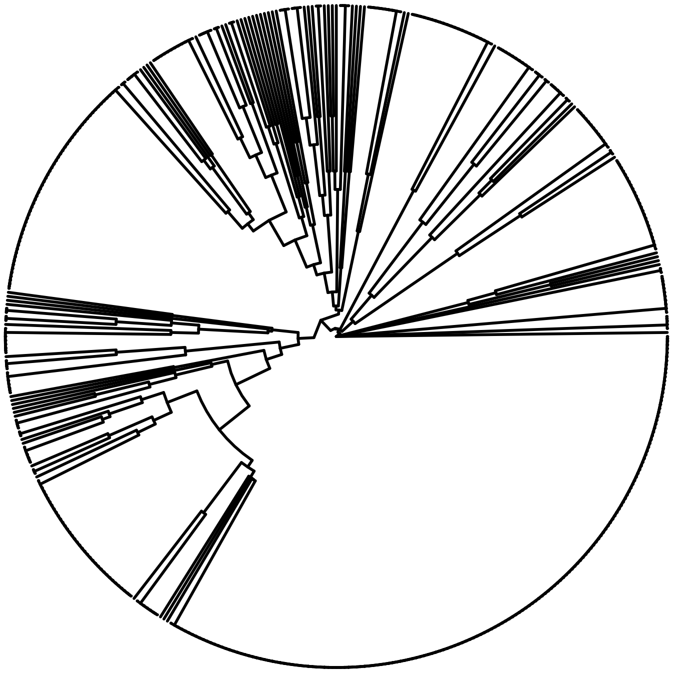

Add further information to the tree. Get manual color scale to have a clearer distinction between Germany and France (most objects).

```
colourCount = length(unique(objects$country))
getPalette = colorRampPalette(brewer.pal(11, "Paired"))

final.tree <- ggtree.plot %<+% objects +
  geom_tippoint(aes(colour = country, angle = angle), alpha = 1,
                show.legend = TRUE, size = 1) +
  geom_tiplab(aes(colour = country, angle = angle, label = object_id),
              hjust = -0.2 , alpha = 1, size = 1) +
  scale_color_manual(values = getPalette(colourCount)) +
  theme(legend.position = "right")
print(final.tree)
```

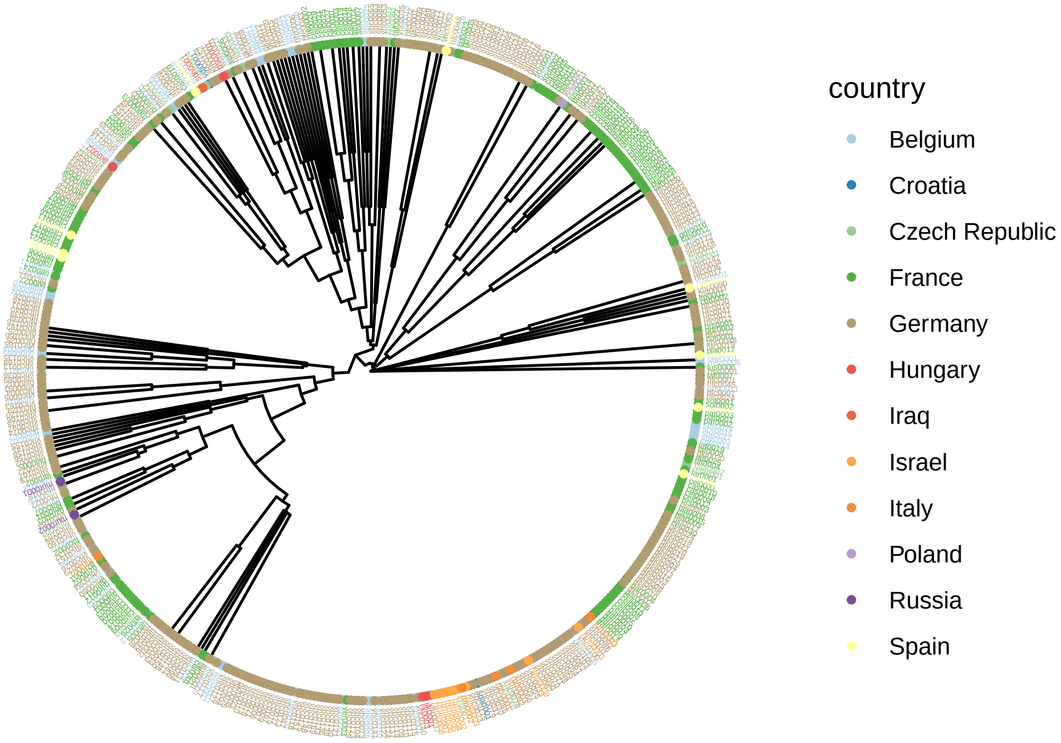

Add "heatmap" illustrating actual vector values for sign type presence. Select columns with particular sign types (the plot is very crowded with all 30 sign types in).

```
signs.select <- select(objects, line, notch, vulva, cross) # select the sign types
# to be displayed: line, obline, radline, dashline, circumline, notch, obnotch,
# radnotch, circumnotch, dot, cupule, cross, rhombus, grid, hatching, zigzag,
# zigzagrow, rectangle, hashtag, maccaroni, v, circumspiral, vulva, anthropomorph,
# zoomorph, paw, concenline, pinleft, pinright, star
signs.select[] <- lapply(signs.select, gsub, pattern = c("1"),
                         replacement = c("present"), fixed = TRUE) # replace the
# integers (0, 1) by factors (absent, present) for plotting
signs.select[] <- lapply(signs.select, gsub, pattern = c("0"),
                         replacement = c("absent"), fixed = TRUE)


tree.heatmap <- gheatmap(final.tree, signs.select, offset = 0.05, width = 0.5,
                         color = "grey",
                         font.size = 4, colnames_angle = -90, hjust = 1.1,
                         colnames_position = "bottom") +
                         scale_fill_manual(values = c("white", "black"))
print(tree.heatmap)
```

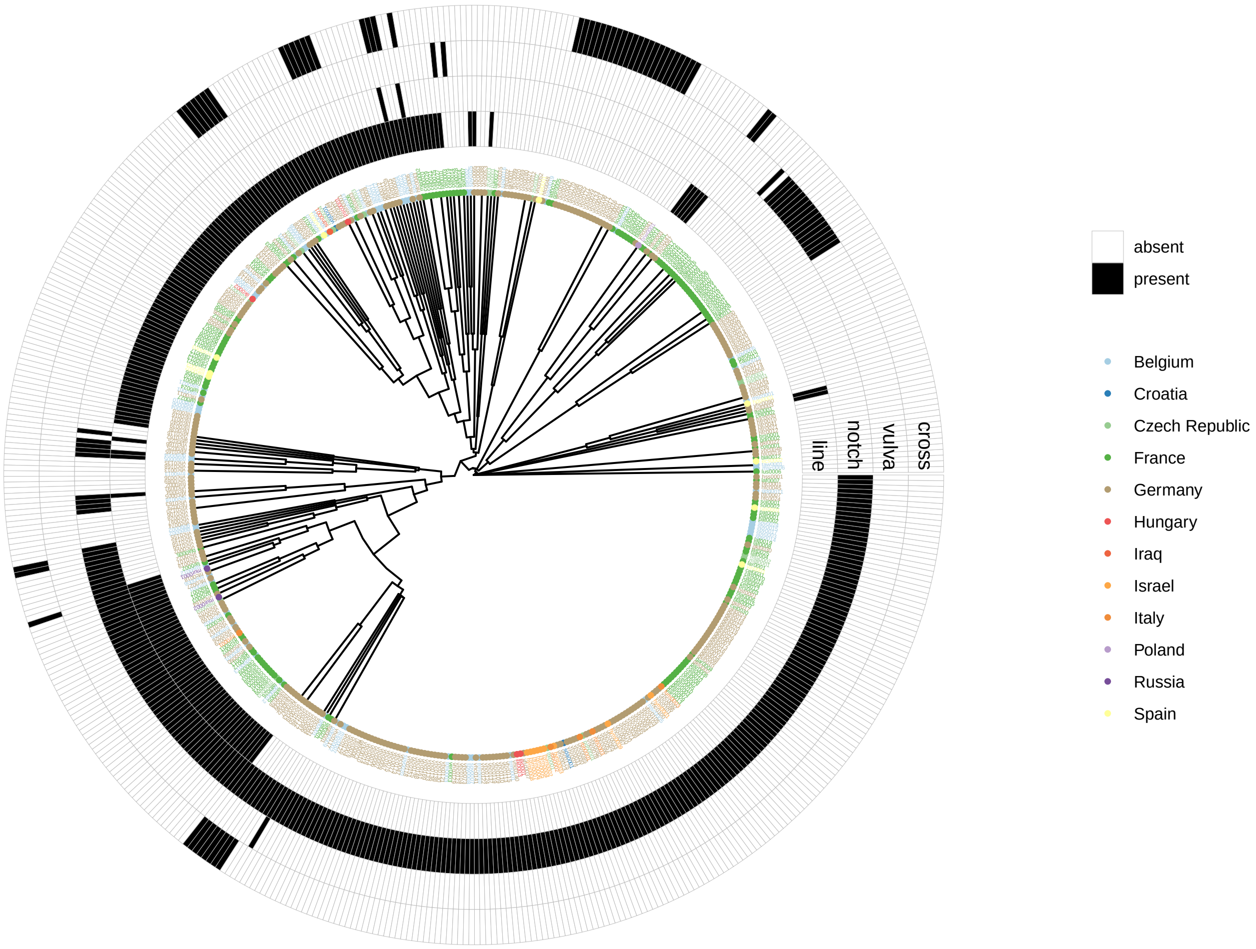

Safe tree as pdf.

```
ggsave("Figures/Figure_TreeHeatmap.pdf", tree.heatmap, dpi = 300, scale = 1,
       device = cairo_pdf)
```
