# Supplementary material for: SignBase, a collection of geometric signs on mobile objects in the Paleolithic
Source: Sci Data. 2020 Oct 23;7:364. doi: 10.1038/s41597-020-00704-x (PMC7585433; doi:10.1038/s41597-020-00704-x)
Supplement: Supplementary file 5 [file 41597_2020_704_MOESM5_ESM.pdf]

SignBase: Jaccard distances and UPGMA trees - worked example

Chris Bentz

July 29, 2020

Load libraries

Load the following packages. If these are not yet installed use install.packages("") to install them.

```
library(stringdist)
library(phangorn)
library(ggtree)
library(RColorBrewer)
library(dplyr)
```

ggtree installation

ggtree is not a standard package at CRAN. In the version of R used here (3.4.4) it has to be installed using the following code.

```
# source("https://bioconductor.org/biocLite.R")
# BiocInstaller::biocLite(c("ggtree"))
```

Load data

Run this code to load the file with data on Aurignacian objects.

```
objects.example <- read.csv("Data/signBase_exampleObjects.csv")
nrow(objects.example)
```

```
## [1] 3
```

Remove sign type column "other".

```
objects.example <- subset(objects.example, select = -c(other))
```

Jaccard distance matrix between pairs of objects based on sign type presences

Initialize empty vector.

```
jaccVec <- c()
```

Create an alphabet to represent absence/presence of a given sign type.

```
alphabet <- c("a","b","c","d","e","f","g","h","i","j",
              "k","l","m","n","o","p","q","r","s","t",
              "u","v","w","x","y","z", "A", "B", "C", "D",
              "E", "F", "G", "H", "I", "J", "K", "L", "M", "N")
```

Draw characters from the alphabet to represent given number of sign types.

```
sign.alphabet <- sample(alphabet, ncol(objects.example[, 21:ncol(objects.example)]))
```

Use for-loop to replace 1s (sign type present) by respective character. This is necessary to then later apply the function stringdistmatrix() to the strings. There is probably a simpler solution to compute Jaccard distances directly on binary vectors, but I was not able to find a ready-made and robust function for this.

```
for (i in 1:nrow(objects.example)) {
  jaccString <- ""
  for (j in 20:ncol(objects.example)) {
    if (objects.example[i,j] == 1) {
      jaccString <- paste(jaccString, sign.alphabet[j-19], sep = "")
    } else {
      jaccString <- jaccString
    }
  }
  jaccVec <- append(jaccVec, jaccString)
}
```

Apply Jaccard distance function to create matrix of pairwise distances between objects.

```
signDistMatrix.example <- stringdistmatrix(jaccVec, jaccVec, method = "jaccard")
print(signDistMatrix.example)
```

```
##           [,1]      [,2]      [,3]
## [1,] 0.0000000 0.6666667 0.5714286
## [2,] 0.6666667 0.0000000 0.8888889
## [3,] 0.5714286 0.8888889 0.0000000
```

UPGMA tree based on Jaccard distance matrix

Create UPGMA tree based on the distance matrix based on sign type presences

```
signTree.example <- upgma(signDistMatrix.example)
```

Create first column in data frame "signs" with running numbers from 1 to the number of objects. This is needed as ggtree takes the numbers in the first column as identifier for tip values (for whatever reason).

```
tip.id <- 1:nrow(objects.example)
objects.example <- cbind(tip.id, objects.example)
```

Plot ggtree with just tree branches. Branch lengths are displayed.

```
ggtree.example <- ggtree(signTree.example) +
  geom_text(aes(x = branch, label = round(branch.length, digits = 2)),
            vjust = -.5, size = 3)
print(ggtree.example)
```

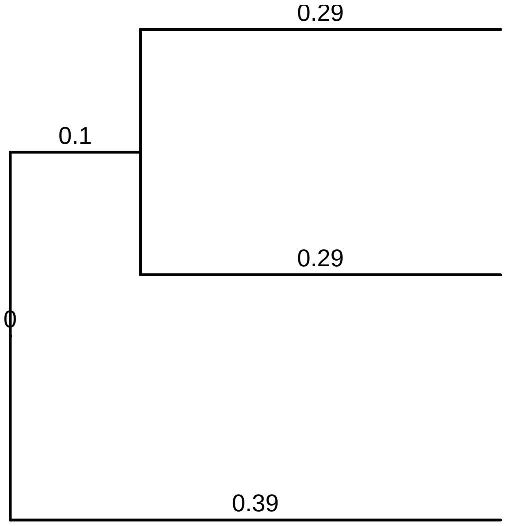

Add further information to the tree, i.e. colour per country.

```
final.tree.example <- ggtree.example %<+% objects.example +
  geom_tippoint(aes(colour = country), alpha = 1, show.legend = TRUE, size = 3) +
  geom_tiplab(aes(colour = country, label = object_id), hjust = -0.2 ,
              alpha = 1, size = 3) +
  geom_text(aes(x = branch, label = round(branch.length, digits = 2)),
            vjust = -.5, size = 3)
```

Add "heatmap" illustrating actual vector values for sign type presence. Select columns with particular sign types (the plot is very crowded with all 30 sign types in).

```
signs.select <- select(objects.example, line, obline, radline, dashline,
                       concenline, dot, cross, v, hatching, zigzagrow) # choose only
# the ones represented on at least one of these three objects
signs.select[] <- lapply(signs.select, gsub, pattern = c("1"),
                         replacement = c("present"), fixed = TRUE) # replace the
# integers (0, 1) by factors (absent, present) for plotting
signs.select[] <- lapply(signs.select, gsub, pattern = c("0"),
                         replacement = c("absent"), fixed = TRUE)

tree.heatmap.example <- gheatmap(final.tree.example, signs.select,
                                 offset = 0.07, width = 0.5, color = "dark grey",
                                 font.size = 3, colnames_angle = 40, hjust = 0,
                                 colnames_position = "bottom") +
  scale_fill_manual(values = c("white", "black"))
print(tree.heatmap.example)
```

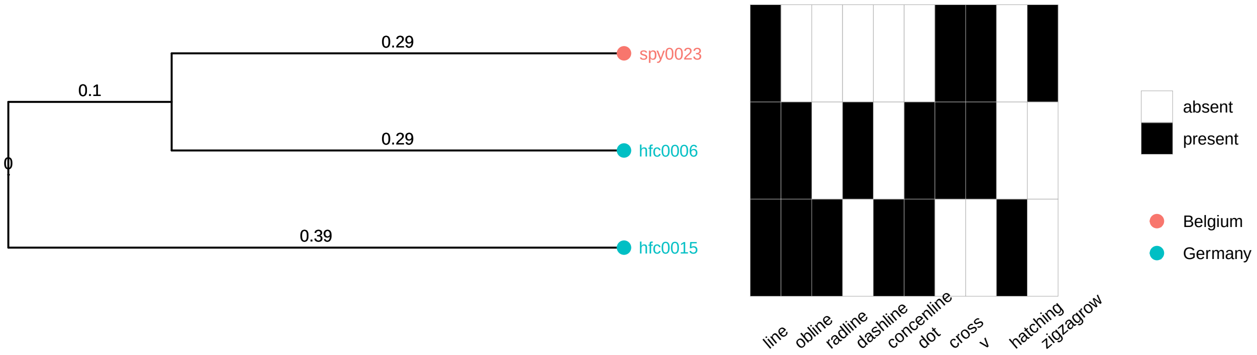

Safe tree as pdf.

```
ggsave("Figures/Figure_TreeHeatmap_example.pdf", tree.heatmap.example, dpi = 300,
       scale = 1, device = cairo_pdf)
```
