# Supplementary material for: SignBase, a collection of geometric signs on mobile objects in the Paleolithic
Source: Sci Data. 2020 Oct 23;7:364. doi: 10.1038/s41597-020-00704-x (PMC7585433; doi:10.1038/s41597-020-00704-x)
Supplement: Supplementary file 6 [file 41597_2020_704_MOESM6_ESM.pdf]

SignBase: Coding Evaluation

Chris Bentz

July 29, 2020

Load Libraries

```
library(ggplot2)
library(reshape2)
library(dplyr)
library(gridExtra)
library(irr)
```

Load files

TestCoding_000.csv (loaded as object test0) is the original file coded by the SignBase team and used for analyses in the article. TestCoding_001.csv to TestCoding004.csv are codings by other researchers.

```
test0 <- read.csv("Data/Tests/TestCoding_000.csv", header = T)
head(test0)
```

```
##   access_id object_id techno_complex       site_name           location
## 1       226   gdr0005    Aurignacien Grotte du Renne      Arcy-sur-Cure
## 2         3   aur0001    Aurignacien        Aurignac           Aurignac
## 3         8   bla0005    Aurignacien       Blanchard Sergeac (Dordogne)
## 4        59   bla0015    Aurignacien       Blanchard Sergeac (Dordogne)
## 5        63   bla0019    Aurignacien       Blanchard Sergeac (Dordogne)
## 6        16   cas0001    Aurignacien        Castanet Sergeac (Dordogne)
##   country longitude latitude line obline radline dashline circumline notch
## 1  France  3.740833 47.60361    0      0       0        0          0     0
## 2  France  0.880000 43.22000    1      0       0        0          0     1
## 3  France  1.100000 45.00000    0      0       0        0          0     1
## 4  France  1.100000 45.00000    1      0       0        0          0     0
## 5  France  1.100000 45.00000    0      0       0        0          0     0
## 6  France  1.120000 44.99000    1      0       0        0          0     1
##   obnotch radnotch circumnotch dot cupule cross rhombus grid hatching
## 1       0        0           0   0      0     1       0    0        0
## 2       0        0           0   0      0     0       0    0        0
## 3       0        0           0   1      0     0       0    0        0
## 4       0        0           0   1      0     0       0    0        0
## 5       0        0           0   1      0     0       0    0        0
## 6       0        0           0   0      0     0       0    0        0
##   zigzag zigzagrow rectangle hashtag maccaroni v circumspiral vulva
## 1      0         0         0       0         0 0            0     0
## 2      0         0         0       0         0 0            0     0
## 3      0         0         0       0         0 0            0     0
## 4      0         0         0       0         0 0            0     0
## 5      0         0         0       0         0 0            0     0
## 6      0         0         0       0         0 0            0     0
##   anthropomorph zoomorph paw concenline pinleft pinright star other
```

```
## 1              0         0   0          0       0         0    0     0
## 2              0         0   0          0       0         0    0     0
## 3              0         0   0          0       0         0    0     0
## 4              0         0   0          0       0         0    0     0
## 5              0         0   0          0       0         0    0     0
## 6              0         0   0          0       0         0    0     0
```

```
test1 <- read.csv("Data/Tests/TestCoding_001.csv", header = T)
test2 <- read.csv("Data/Tests/TestCoding_002.csv", header = T)
test3 <- read.csv("Data/Tests/TestCoding_003.csv", header = T)
test4 <- read.csv("Data/Tests/TestCoding_004.csv", header = T)
```

Pre-Processing

Select relevant columns of the data files. That is, the object identfiers in column 2, and the sign type codings in columns 9 to 39.

```
test0.short <- subset(test0, select = c(2, 9:39))
test1.short <- subset(test1, select = c(2, 9:39))
test2.short <- subset(test2, select = c(2, 9:39))
test3.short <- subset(test3, select = c(2, 9:39))
test4.short <- subset(test4, select = c(2, 9:39))
```

Transform the resulting data frames into long format. The column object_identifier is used as id variable for formatting to long format.

```
test0.long <- melt(test0.short)
test1.long <- melt(test1.short)
test2.long <- melt(test2.short)
test3.long <- melt(test3.short)
test4.long <- melt(test4.short)
```

Calculate Pairwise Agreement

The coding agreement is here calculated simply as the percentage of agreed codings between coders (either both choose 0, i.e. sign type absent, or both choose 1, i.e. sign type present). This is done pairwise for the SignBase coding (test0) with the respective coding according to the other coders (test1, test2, test3, test4). The function agree() in the irr package is used here.

```
# agreement with test1
coder01.df <- cbind(test0.long$value, test1.long$value)
agree(coder01.df)
```

```
##  Percentage agreement (Tolerance=0)
##
##  Subjects = 930
##    Raters = 2
##   %-agree = 93.2
```

```
percent01 <- round(agree(coder01.df)$value, 0) # the percentage value is safed for
# plotting later in the tile plots

# agreement with test2
```

```
coder02.df <- cbind(test0.long$value, test2.long$value)
agree(coder02.df)
```

```
##  Percentage agreement (Tolerance=0)
##
##  Subjects = 930
##    Raters = 2
##   %-agree = 94
```

```
percent02 <- round(agree(coder02.df)$value, 0)

#agreement with test3
coder03.df <- cbind(test0.long$value, test3.long$value)
agree(coder03.df)
```

```
##  Percentage agreement (Tolerance=0)
##
##  Subjects = 930
##    Raters = 2
##   %-agree = 90.8
```

```
percent03 <- round(agree(coder03.df)$value, 0)

#agreement with test4
coder04.df <- cbind(test0.long$value, test4.long$value)
agree(coder04.df)
```

```
##  Percentage agreement (Tolerance=0)
##
##  Subjects = 930
##    Raters = 2
##   %-agree = 93.3
```

```
percent04 <- round(agree(coder04.df)$value, 0)
```

Calculate Cohen's Kappa

Cohen's Kappa is here calculated pairwise as above, i.e. between the original SignBase coding (test0) and all the other codings (test1-test4). The data frames with codings from above can here be used again. We use the function kappa2() of the irr package.

```
#Cohen's Kappa of test0 with with test1-test4
kappa2(coder01.df)
```

```
##  Cohen's Kappa for 2 Raters (Weights: unweighted)
##
##  Subjects = 930
##    Raters = 2
##     Kappa = 0.443
##
##         z = 13.6
##   p-value = 0
```

```
kappa01 <- round(kappa2(coder01.df)$value, 2) # safe value for plotting later
```

```r
kappa2(coder02.df)
```

```
##  Cohen's Kappa for 2 Raters (Weights: unweighted)
##
##  Subjects = 930
##    Raters = 2
##     Kappa = 0.44
##
##         z = 13.4
##   p-value = 0
```

```r
kappa02 <- round(kappa2(coder02.df)$value, 2)

kappa2(coder03.df)
```

```
##  Cohen's Kappa for 2 Raters (Weights: unweighted)
##
##  Subjects = 930
##    Raters = 2
##     Kappa = 0.29
##
##         z = 8.97
##   p-value = 0
```

```r
kappa03 <- round(kappa2(coder03.df)$value, 2)

kappa2(coder04.df)
```

```
##  Cohen's Kappa for 2 Raters (Weights: unweighted)
##
##  Subjects = 930
##    Raters = 2
##     Kappa = 0.345
##
##         z = 10.6
##   p-value = 0
```

```r
kappa04 <- round(kappa2(coder04.df)$value, 2)
```

Visualization of Coding Agreement

Create a data frame with "agreement" between pairwise comparisons of the original SignBase codings with the respective other codings. Agreement is here simply defined as the difference between the codings (0 and 1) taken as an absolute value, i.e. whenever the codings disagree for a given object and sign type we get 1, whenever they agree we get 0. Data frames with these agreement vectors are created for plotting.

```r
#test0 and test1
agreement01 <- subset(test0.long, select = c("object_id", "variable"))
agreement01$value <- abs(test0.long$value-test1.long$value)

#test0 and test2
agreement02 <- subset(test0.long, select = c("object_id", "variable"))
agreement02$value <- abs(test0.long$value-test2.long$value)
```

```r
#test0 and test3
agreement03 <- subset(test0.long, select = c("object_id", "variable"))
agreement03$value <- abs(test0.long$value-test3.long$value)

#test0 and test4
agreement04 <- subset(test0.long, select = c("object_id", "variable"))
agreement04$value <- abs(test0.long$value-test4.long$value)
```

This code produces tile plots for the original coding by the SignBase team, and then tile plots for the agreement/disagreement with the other coders.

```r
test0.plot <- ggplot(test0.long, aes(variable, object_id)) +
  geom_tile(aes(fill = value), colour = "white") +
  scale_fill_gradient(low = "light grey", high = "black") +
  ggtitle("SignBase Coding") +
  scale_x_discrete(expand = c(0, 0)) +
  scale_y_discrete(expand = c(0, 0)) +
  theme(legend.position = "none",
        axis.text.x = element_text(angle = 90, hjust = 1, vjust = 0.4),
        axis.title.x = element_blank())

agreement01.plot <- ggplot(agreement01, aes(variable, object_id)) +
  geom_tile(aes(fill = value), colour = "white") +
  scale_fill_gradient(low = "green", high = "red") +
  ggtitle("Agreement with Coder 1") +
  scale_x_discrete(expand = c(0, 0)) +
  scale_y_discrete(expand = c(0, 0)) +
  annotate("text", x = 20, y = 6, size = 7, colour = "black",
           label = paste("Agree.:", paste(percent01, "%"), sep = ' ')) +
  annotate("text", x = 20, y = 3, size = 7, colour = "black",
           label = paste("Kappa:", kappa01, sep = ' ')) +
  theme(legend.position = "none",
        axis.text.x = element_text(angle = 90, hjust = 1, vjust = 0.4),
        axis.title.x = element_blank(),
        axis.title.y = element_blank(),
        axis.text.y = element_blank(),
        axis.ticks.y = element_blank())

agreement02.plot <- ggplot(agreement02, aes(variable, object_id)) +
  geom_tile(aes(fill = value), colour = "white") +
  scale_fill_gradient(low = "green", high = "red") +
  ggtitle("Agreement with Coder 2") +
  scale_x_discrete(expand = c(0, 0)) +
  scale_y_discrete(expand = c(0, 0)) +
  annotate("text", x = 20, y = 6, size = 7, colour = "black",
           label = paste("Agree.:", paste(percent02, "%"), sep = ' ')) +
  annotate("text", x = 20, y = 3, size = 7, colour = "black",
           label = paste("Kappa:", kappa02, sep = ' ')) +
  theme(legend.position = "none",
        axis.text.x = element_text(angle = 90, hjust = 1, vjust = 0.4),
        axis.title.x = element_blank(),
        axis.title.y = element_blank(),
        axis.text.y = element_blank(),
        axis.ticks.y = element_blank())
```

```
agreement03.plot <- ggplot(agreement03, aes(variable, object_id)) +
  geom_tile(aes(fill = value), colour = "white") +
  scale_fill_gradient(low = "green", high = "red") +
  ggtitle("Agreement with Coder 3") +
  scale_x_discrete(expand = c(0, 0)) +
  scale_y_discrete(expand = c(0, 0)) +
  annotate("text", x = 20, y = 6, size = 7, colour = "black",
           label = paste("Agree.:", paste(percent03, "%"), sep = ' ')) +
  annotate("text", x = 20, y = 3, size = 7, colour = "black",
           label = paste("Kappa:", kappa03, sep = ' ')) +
  theme(legend.position = "none",
        axis.text.x = element_text(angle = 90, hjust = 1, vjust = 0.4),
        axis.title.x = element_blank(),
        axis.title.y = element_blank(),
        axis.text.y = element_blank(),
        axis.ticks.y = element_blank())

agreement04.plot <- ggplot(agreement04, aes(variable, object_id)) +
  geom_tile(aes(fill = value), colour = "white") +
  scale_fill_gradient(low = "green", high = "red") +
  ggtitle("Agreement with Coder 4") +
  scale_x_discrete(expand = c(0, 0)) +
  scale_y_discrete(expand = c(0, 0)) +
  annotate("text", x = 20, y = 6, size = 7, colour = "black",
           label = paste("Agree.:", paste(percent04, "%"), sep = ' ')) +
  annotate("text", x = 20, y = 3, size = 7, colour = "black",
           label = paste("Kappa:", kappa04, sep = ' ')) +
  theme(legend.position = "none",
        axis.text.x = element_text(angle = 90, hjust = 1, vjust = 0.4),
        axis.title.x = element_blank(),
        axis.title.y = element_blank(),
        axis.text.y = element_blank(),
        axis.ticks.y = element_blank())
```

Arrange all the tile maps into one plot.

```
eval.plot <- grid.arrange(test0.plot, agreement01.plot, agreement02.plot,
                          agreement03.plot, agreement04.plot, ncol = 5,
                          widths = c(2.5, 2, 2, 2, 2))
```

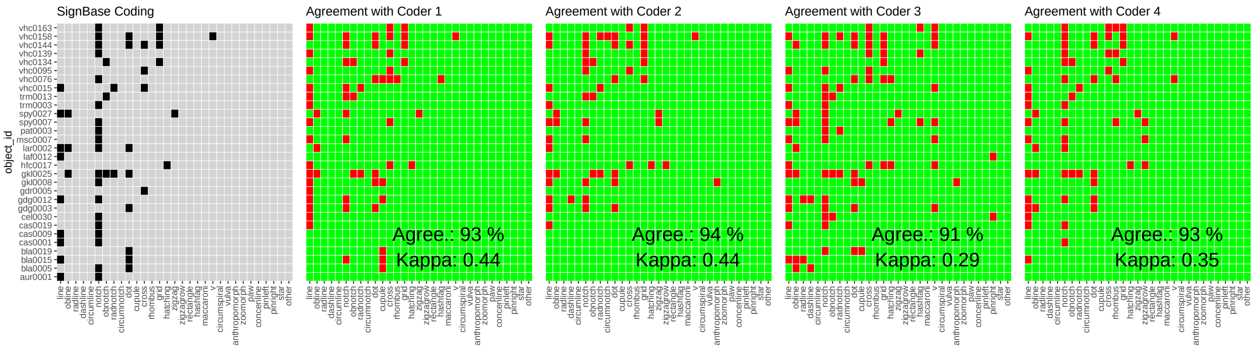

Safe tile plots as pdf.

```r
ggsave("Figures/Figure_CodingEvaluation.pdf", eval.plot, dpi = 300,
       scale = 1, device = cairo_pdf)
```
